# Systematic Review and Network Meta-Analysis on Treating Hormone Receptor-Positive Metastatic Breast Cancer After CDK4/6 Inhibitors

**DOI:** 10.3390/curroncol32010053

**Published:** 2025-01-20

**Authors:** Neha Pathak, Abhenil Mittal, Sudhir Kumar, Chitrakshi Nagpal, Eitan Amir, Partha Haldar, Bharath B. Gangadharaiah, Akash Kumar, Ashutosh Mishra, Atul Batra

**Affiliations:** 1 Division of Medical Oncology & Hematology, Department of Medicine, Princess Margaret Cancer Centre, Toronto, ON M5G 2M9, Canada; 2 Department of Medicine, University of Toronto, Toronto, ON M5S 1A1, Canada; 3 Health Sciences North, Northern Ontario School of Medicine (NOSM U), Sudbury, ON P3E 5J1, Canada; 4 Odette Cancer Center, Sunnybrook Health Sciences Center, Toronto, ON M4N 3M5, Canada; 5 Department of Medical Oncology, All India Institute of Medical Sciences, New Delhi 110029, India; chitrakshinagpal11.jan20@aiims.edu (C.N.);; 6 Centre for Community Medicine, All India Institute of Medical Sciences, New Delhi 110029, India; 7 Department of Surgical Oncology, All India Institute of Medical Sciences, New Delhi 110029, India

**Keywords:** metastatic breast cancer, CDK4/6 inhibitor, hormone receptor-positive cancer, SERD, HER2-low breast cancer, AKT inhibitor

## Abstract

Introduction: The optimal treatment of estrogen receptor-positive (ER +) metastatic breast cancer (MBC) after progression on cyclin-dependent 4/6 kinase inhibitors (CDK4/6i) is unknown. Methods: We conducted a systematic review and network meta-analysis (NMA) of phase-II/-III randomized trials of ER + MBC post CDK4/6i + ET progression. We calculated the hazard ratio (HR) for progression-free survival (PFS) and overall survival (OS) using generic inverse variance and odds ratios (ORs) using the Mantel–Haenszel method for adverse events (AEs) with Review-Manager version-5.4. NMA was executed using WINBUGS (Microsoft Excel). Three molecular subgroups were analyzed: HER2-low, PI3K/AKT/mTOR, and the ESR1 mutation subgroup for selective estrogen receptor degrader (SERD). Results: A total of 14 studies were included. In the HER2-low group, Sacituzumab govitecan and trastuzumab deruxtecan had a similar efficacy (HR, 95% CI): PFS (0.98; 0.63–1.43) and OS (1.08; 0.76–1.55). In PI3K/AKT/mTOR-altered cases, capivasertib was superior to alpelisib PFS (0.77; 0.53–1.12), and OS (0.80; 0.48–1.35). SERDs had worse PFS and OS versus ongoing CDK 4/6i (ribociclib). Conclusion: No therapy emerged as the unequivocal choice in the post-CDK 4/6i domain in unselected subgroups. In the HER2-low population, a similar efficacy and different toxicity spectrum was seen. In AKT-altered tumors, capivasertib was less toxic than alpelisib. PROSPERO ID: CRD4202236412.

## 1. Introduction

Cyclin-dependent 4/6 kinase inhibitors (CDK4/6i) have emerged as the standard first-line treatment in patients with endocrine receptor (estrogen and/or progesterone receptor-positive; ER)-positive, human epidermal growth factor receptor-2 (HER2)-negative metastatic breast cancer (MBC) [1,2]. Several randomized controlled trials (RCTs) and real-world studies have demonstrated the efficacy and safety of CDK4/6i (palbociclib, abemaciclib, and ribociclib) with endocrine therapy (ET) in the first- and second-line setting [3,4,5,6,7,8,9,10].

While first-line therapy is an acceptable standard of care across the globe, the choice of therapy after progression on CDK 4/6i varies significantly [11,12,13]. This includes second-line ET, chemotherapy, and phosphatidylinositol-3-kinase (PI3K) inhibitors like alpelisib [14], as well as AKT inhibitors like Capivasertib [15] and oral selective estrogen receptor degraders or down-regulators (SERDs) such as Elacestrant [16] among other options. In subsequent lines, antibody–drug conjugates (ADCs) such as trastuzumab deruxtecan for patients with HER-2 low tumors [17] and sacituzumab govitecan may be used [18].

Furthermore, continuing CDK 4/6i beyond progression with change in the partner ET has also been evaluated [19,20,21,22]. However, this has resulted in varying results and modest benefits [21,22,23,24]. A better understanding of resistance mechanisms is required to identify patients who may benefit from this approach [25].

Despite several treatment options after progression on CDK4/6i, the optimal treatment remains unknown, given the lack of direct comparisons between different agents and limited exposure to CDK4/6i in the first line in these studies. To address this knowledge gap, we designed this systematic review and a network meta-analysis (NMA) of therapeutic options after first-line CDK 4/6i in ER+ HER2- MBC. Recognizing that ER + MBC is a heterogenous disease with multiple molecularly driven subgroups (HER2-low, AKT/PI3K/mTOR, mESR1), we planned to assess the efficacy and toxicity of drugs in each group, with the aim of identifying the most suitable agent in each class. We hypothesized that indirect comparisons using a network meta-analysis for each subgroup would allow for better benefit–risk comparisons against the current standard of care for that molecular pathway, if known.

## 2. Materials and Methods

This study was conducted in accordance with the Preferred Reporting Items for Systematic Reviews and Meta-analyses (PRISMA) guidelines [26] and relevant extension statements for incorporating network meta-analyses of healthcare interventions [27]. The protocol was registered prospectively on PROSPERO (CRD42022364121).

### 2.1. Selection Criteria

The studies included were phase-II/-III randomized controlled trials that enrolled patients with ER+/HER2-negative MBC in the second-/subsequent-line setting after progression on a CDK4/6i-based first-line treatment. Survival outcomes (progression-free survival [PFS] and overall survival [OS]) must have been reported, and the full text published, to allow for risk of bias assessment.

Studies including only triple-negative breast cancer or HER2-positive breast cancer were excluded. Single-arm prospective studies were collected for systematic review but not for quantitative analysis. Studies published in abstract form only were not included.

### 2.2. Information Sources

We searched MEDLINE, Embase, the Cochrane Library and clinicaltrials.gov without any time period or language restrictions. In addition, conference abstracts were reviewed from 2017 to 2022 from the leading oncologic conferences: American Society of Clinical Oncology (ASCO), European Society of Medical Oncology (ESMO), and San Antonio Breast Cancer Symposium (SABCS). The reference lists in relevant articles were scanned. The final search of all databases was run on 29 September 2022. Thereafter, only updates to relevant articles were obtained.

### 2.3. Search Strategy

The search strategy was tailored to the database being searched. MESH terms included “breast neoplasms”, “cyclin-dependent kinase inhibitors”, “hormone”, and the text words “palbociclib”, “abemaciclib”, and “ribociclib”, all combined with appropriate Boolean operators. Details of the search strategy used for each database can be found in Supplementary Materials File S1.

Authors were contacted for six studies [14,28,29,30,31,32], and for two studies, database repositories [17,32] were emailed for the required survival data from 30 January 2023 to 7 February 2023. Database sharing was denied via email on 30 January 2023 for the DESTINY-Breast04 study [17] as the follow-up was ongoing and for Ipatunity-130 on 27 February 2023 due to legal/contractual compliance issues [32]. For another study, the corresponding author declined our request to share relevant data, as a small number of patients treated with CDK 4/6i (n = 15) were not analyzed separately [28]. We did not receive a reply for the remaining studies.

### 2.4. Data Extraction Process and Data Items

Relevant data consisting of the study identification details, first author name, year of publication, phase of the study, number of participants of interest, intervention and control arms, and results from randomized controlled trials were extracted by two authors (SK and NP) separately and then compiled. Any discrepancy was resolved through discussion with other authors (AB and PH).

### 2.5. Outcomes

Efficacy outcomes for each subgroup were assessed using OS, defined as the time from randomization on the respective trial to death due to any cause across included studies and PFS, measured from randomization to the progression of disease or death due to any cause as defined by individual studies and toxicity. This was carried out in the relevant molecularly driven subgroups: HER2-low, PI3K/AKT/mTOR pathway, and mESR1.

Toxicities were quantified as hematologic vs. non-hematologic grade ≥ 3 adverse events for all studies vs. ribociclib and, further, using the twelve most frequent adverse events (AEs) [33] reported for cancer drugs, as well as some relevant, drug class-specific adverse events, such as hyperglycemia (AKT/mTOR/PI3K pathway-altering drugs), for subgroup analyses. AEs were measured and reported by standard methods such as the common terminology criteria for adverse events (CTCAE).

As an exploratory analysis, in addition to molecular pathway-driven group-wise comparisons, we also conducted a PFS and AE comparison of all agents against the strategy of switching CDK4/6i + ET as carried out in the MAINTAIN trial [19]. We hypothesized that, with the knowledge that the strategy of changing the ET and type of CDK4/6i after progression on CDK4/6i +ET led to modest benefits, any effective therapy should be superior to this option. This was hypothesized with consensus from all authors.

### 2.6. Risk of Bias

Eligible RCTs were assessed using the revised Cochrane risk of bias tool for randomized trials (RoB 2) [34] under five headings: bias from the randomization process (selection bias), bias due to deviation from intended interventions, bias due to missing outcome data (attrition bias), bias in the measurement of outcomes (detection bias), and bias in the selection of reported result (reporting bias). Studies were graded by CN with regard to bias as low, high, or having some concerns or no information. Any discrepancies were resolved through discussion with AB.

### 2.7. Statistical Analysis

Multiple interventions were available for the treatment of ER + HER2- MBC after exposure to CDK 4/6i. Trials were divided into subgroups based on the mechanism of action of the experimental drug. However, most of these studies contained a mix of both CDK 4/6i-exposed and -unexposed patients. The results for OS after CDK4/6i exposure were available for three studies only: TROPiCS-02 [35], EMERALD [16], and VERONICA [36]. OS comparisons were possible for trastuzumab deruxtecan, and sacituzumab govitecan (HER2-low population) for the HER2-low population irrespective of prior CDK 4/6i [17,35] and between alpelisib and capivasertib (all PI3K/AKT pathway-altered groups) [15,37].

We performed PFS comparisons of all studies by NMA. For some studies [14,15,28,29,31,32] mainly involving the PI3K/AKT/mTOR pathway, the HR of the whole population/targeted pathway population was considered, as some of these [14,28] had too few patients exposed to CDK 4/6i for us to make meaningful comparisons. Comparisons for efficacy using PFS were made against alpelisib for all agents acting through the PI3K/AKT/mTOR pathway. A network meta-analysis for PFS in the HER2-low population was performed for trastuzumab deruxtecan and sacituzumab govitecan. Similarly, a comparison between elacestrant and amcenestrant was also performed in the ESR1-mutant population.

For AEs, the OR was calculated from data for the whole study population, as the risk of an AE was assumed to not be different for patients exposed to CDK 4/6i vs. those who were not.

There were no OS results available from the MAINTAIN study [19], which evaluated the effects of continuing another CDK4/6i (ribociclib) after progression on a CDK4/6i, with ET. We performed PFS comparisons of all agents with a ribociclib switch, as well as grade ≥3 AE (hematological and non-hematological).

For the outcomes for OS, PFS, and AEs, we calculated the hazard ratio (HR) for PFS and odds ratio (OR) and respective 95% confidence interval (95%CI) as appropriate, using Review Manager Version 5.4 (The Cochrane Collaboration, Copenhagen, Denmark). The generic inverse variance was used to calculate HR and the Mantel–Haenszel method was used for the OR. Random effects were employed assuming statistical heterogeneity. Pair-wise comparisons were performed to explore differential efficacy (PFS) and safety (AEs). A network meta-analysis was performed using WINBUGS within Microsoft Excel (Microsoft Corp., Redmond, WA, USA), which utilizes a Bayesian approach [38]. Statistical tests were two-sided, and statistical significance was defined a priori as *p* < 0.05. Data were represented using tables and forest plots.

## 3. Results

### 3.1. Study Selection and Study Characteristics

**Figure 1:**
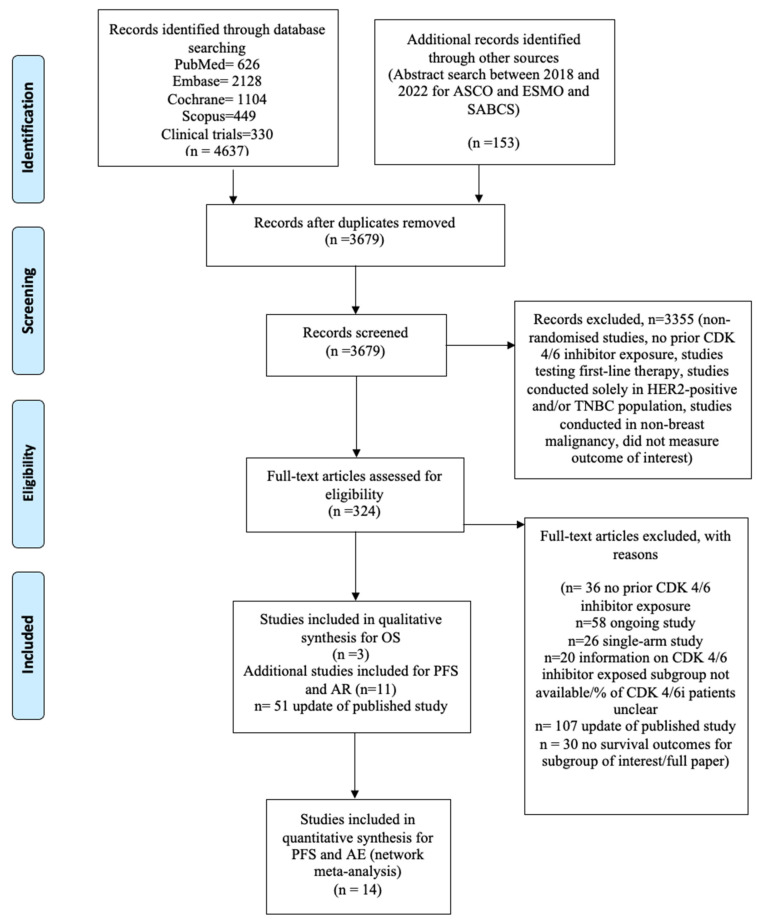
PRISMA flow diagram for the inclusion of studies.

A total of 4790 records were retrieved from the database and gray literature search. After the exclusion of duplicates, 3679 records were retained. Of these, most were excluded for not meeting the search criteria, leaving 330 studies of interest (Figure 1). Only three studies had OS data for patients post CDK 4/6i exposure and included the investigational agents sacituzumab govitecan, venetoclax, and elacestrant against a control [16,36,39]. We had additional OS data for the HER2-low subgroup (sacituzumab govitecan vs. trastuzumab deruxtecan) [17,35] and PI3K/AKT/mTOR (capivasertib vs. alpelisib) [15,37] for the whole population, which contained variable percentages of patients exposed to prior CDK 4/6i. For the endpoints of PFS and AE, data from 11 additional studies were extracted, bringing the total to 14 studies [14,15,17,19,28,29,30,31,32,40,41]. The study designs, outcomes, and baseline characteristics are summarized in Table 1, Table 2 and Table 3. Eight studies were phase-III studies (Table 1); the remainder were phase-II studies [19,29,31,36,40,41]. The agents tested included ribociclib (CDK4/6i switch) with a change in ET, two ADCs (sacituzumab govitecan and trastuzumab deruxtecan), two SERDs (elacestrant and amcenestrant), agents targeting the PI3KCA/AKT/mTOR pathway (alpelisib, taselisib, sapanisertib, capivasertib, and ipatasertib), an aurora A kinase inhibitor (alisertib), an HDAC inhibitor (entinostat), a BCL2 inhibitor (venetoclax), and an IGF 1/2 monoclonal antibody (xentuzumab) (Table 1). No trial had OS as a primary endpoint. Only five agents (Trastuzumab deruxtecan, entinostat, ipatasertib, venetoclax, and amcenestrant) were tested in pre-menopausal patients [17,30,32,36,40]; even then, they constituted a small proportion of the full trial population (Table 3). All studies had over 50% participants with visceral disease, except the xentuzumab study (31%) [41]. All patients were exposed to at least one line of ET. Individual studies defined endocrine resistance among participants. Some specified the proportion of endocrine-resistant patients, and others evaluated mutations that caused endocrine resistance (Table 3). The TROPiCS 02 study [39], which evaluated sacituzumab govitecan, had the most heavily treated patient population (median lines of treatment = 7, median lines of chemotherapy = 3), followed by DESTINY breast 04 [17], assessing Trastuzumab deruxtecan (median lines of treatment =3). Most studies had a heterogeneous population with varying proportions of patients being treated with CDK4/6i in the first-line setting, and only four studies [16,19,36,39] included patients treated with prior CDK4/6i exclusively (Table 3). Four studies had chemotherapy as their comparator arm [17,29,32,39], and the remaining had ET.

Other studies for which the full texts of the papers were not available, or which were single-arm prospective studies testing novel agents in this therapeutic zone, which may form the basis of future RCTs, are summarized in Supplementary Tables S1–S3.

### 3.2. Quality and Risk of Bias

All studies except two (Rugo et al. [39], with details of randomization not mentioned, and Lindeman et al. [36], an open-label trial) had a low risk of selection and performance bias. Most studies were not blinded to outcome assessment. The studies included in the review seemed to have an overall low-to-moderate risk of bias (Supplemental Figure S1a,b).

### 3.3. Therapeutic Subgroup Comparisons

Due to the expected heterogeneity in the patient population, on account of distinct molecular drivers, three distinct groups were created for comparison, as summarized below.

#### 3.3.1. Antibody Drug Conjugates: HER2-Low Population

**Figure 2:**
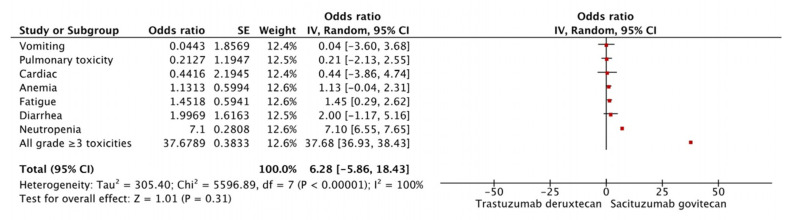
Forest plot of network meta-analysis of grade ≥ 3 adverse events of trastuzumab deruxtecan vs. Sacituzumab govitecan. Red boxes represent the results of each study and the horizontal lines the 95% confidence interval.

Sacituzumab govitecan [39] and Trastuzumab deruxtecan [17] were compared for the HER-2 low population and showed similar efficacy in terms of PFS (HR of 0.98; 95% CI, 0.63–1.43) and OS (HR 1.08; 95% CI, 0.76–1.55). The adverse event profile for the two is depicted in Figure 2. Grade ≥ 3 adverse events, for which data were available for both drugs, were evaluated. Sacituzumab govitecan had more grade ≥ 3 neutropenia (OR 7.10 [95% CI 6.55–7.65]), and higher grade ≥ 3 diarrhea (OR 2.00 [95% CI −1.17–5.16]. There were lower odds of grade ≥ 3 pulmonary toxicity (OR 0.21 [95% CI (−2.13)–2.55]) and vomiting (OR 0.04 [95% CI (−3.6)–3.68], with sacituzumab govitecan. The higher toxicity of Sacituzumab govitecan is driven primarily by neutropenia. Patients in the sacituzumab govitecan study were more heavily treated (median 7, range 3–17) than in the trastuzumab deruxtecan study (median 3, range 1–9).

#### 3.3.2. Drugs Acting on the PI3K/AKT/mTOR Pathway

**Figure 3:**
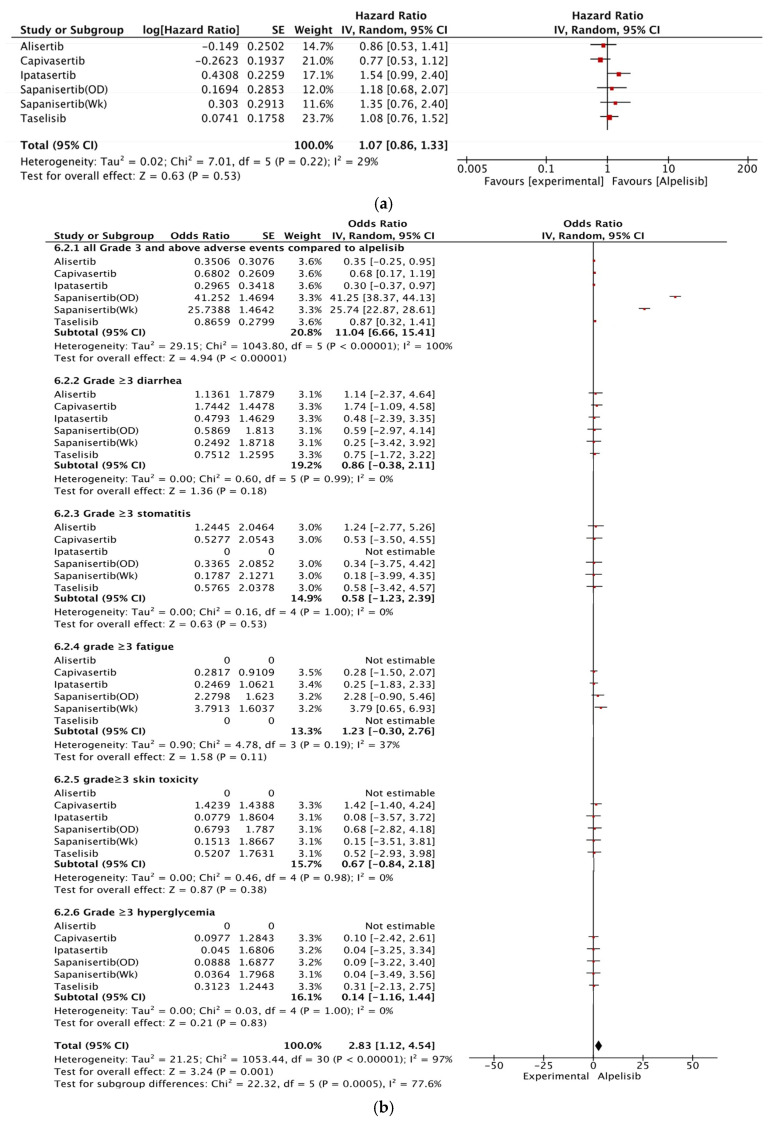
(**a**) Forest plot showing the network meta-analysis of progression-free survival for PI3K/AKT/mTOR pathway-affecting drugs vs. alpelisib. (**b**) Forest plot showing the network meta-analysis of grade ≥ 3 adverse events for PI3K/AKT/mTOR pathway-affecting drugs vs. alpelisib. Red boxes represent the results of each study and the horizontal lines the 95% confidence interval.

Capivasertib [15] performed better than all other agents evaluated [14,28,29,31,32] for PFS (HR, 0.77; 95% CI, 0.53–1.12), although the strength of this result is low as it is derived from a single study per agent. Among capivasertib (AKT pathway-altered) and alpelisib (PI3KCA-mutated), the HR for OS of the pairwise comparison was 0.80 (95% CI, 0.48–1.35), as seen in Figure 3a.

The adverse effects (grade 3 and higher) of this class of drugs have been compared against those of alpelisib (Figure 3b). Common grade ≥ 3 side effects included diarrhea, stomatitis, hyperglycemia, vomiting, fatigue, and skin toxicity. Capivasertib was associated with a higher frequency of diarrhea and skin toxicity but lower hyperglycemia, vomiting, and fatigue. Alpelisib was associated with the maximum frequency of hyperglycemia. Febrile neutropenia was associated exclusively with alpelisib [14].

#### 3.3.3. SERDs

For two SERDs, full publications were available: amcenestrant [40] and elacestrant [16]. For SERD, we chose the CDK4/6i switch with ribociclib as our control. In terms of PFS, elacestrant or amcenestrant, compared to ribociclib, demonstrates a worse HR than continuing ribociclib alone in the mESR1 population (Supplementary Figure S2). Continuing ribociclib in this (mESR1) population in the trial by Kalinsky et al. [19] demonstrated no benefit compared to fulvestrant alone.

The AE analysis is represented in Supplementary Figure S3. Elacestrant had less diarrhea, fatigue, and nausea while having more back pain than amcenestrant. In addition, grade ≥ 3 headaches, arthralgias and liver function abnormalities were seen in elacestrant but not with amcenestrant (not shown in figure).

### 3.4. Exploratory Analysis: Comparison of All Agents with Ribociclib

An NMA for OS could not be performed as there were not enough studies with mature OS data and the absence of subgroup information on HR for OS in CDK 4/6i-treated patients. OS data from the MAINTAIN trial [19], our chosen comparator arm, were also unavailable. Three studies met this criterion, on sacituzumab govitecan [39], venetoclax [36], and elacestrant [16], as they mandated prior CDK 4/6i treatment in their inclusion criteria. These trials are summarized in Table 1, Table 2 and Table 3, above.

The results for the NMA for PFS with ribociclib as a control are depicted in Supplemental Figure S4. When comparing PFS against continuing CDK4/6i alone, no differences were identified indicating similar efficacy: capivasertib HR, 0.88 (95% CI, 0.55–1.40); trastuzumab deruxtecan HR, 0.96 (95% CI, 0.7–1.59); alisertib HR, 0.98 (95% CI, 0.61–1.54); alpelisib, 1.14 (95% CI, 0.72–1.81); sacituzumab govitecan HR, 1.16 (95% CI, 0.79–1.91); taselisib HR, 1.23 (95% CI, 0.78–1.93); elacestrant HR, 1.23 (95% CI, 0.72–2.52); sapanisertib once daily HR, 1.35 (95% CI, 0.82–2.91); sapanisertib weekly HR, 1.54 (95% CI, 1.04–2.95); entinostat HR, 1.52 (95% CI, 0.93–2.93); venetoclax HR 1.65 (95% CI 1.15–2.96); ipatasertib, HR 1.75 (95% CI, 0.88–4.93); amcenestrant HR, 1.84 (95% CI, 0.12–19.06); and xentuzumab HR, 2.09 (95% CI, 0.13–10.54).

An NMA was performed for all hematological (anemia, thrombocytopenia, neutropenia, febrile neutropenia, leukopenia) and non-hematological AEs of grade 3 or higher in comparison with ribociclib in the MAINTAIN study [19].

For hematological grade ≥ 3 AE, all agents tested similar to ribociclib, with odds ratios crossing 1 (Supplemental Figure S5a). For non-hematological grade ≥ 3 toxicities, capivasertib (OR 4.03 [95% CI, 2.78–5.28]), alpelisib (OR 5.76 [95% CI, 4.51–7.01]), taselisib (OR 5.1 [95% CI, 3.83–6.36]) and sapanisertib in both doses, weekly (OR 242.16 [95% CI, 239.06–245.26]) and once daily (OR 151.09 [95% CI, 148.01–154.18]) had greater AEs than ribociclib.

Alisertib (OR 1.30 [95% CI, 0.10–2.70]), trastuzumab deruxtecan (OR 1.39 [95% CI, 0.1–2.67])), Entinostat (OR 1.45 [95% CI, 0.16–2.74]), ipatasertib (OR 1.49 [95% CI, 0.16–2.82]), elacestrant (OR 1.55 [95% CI, 0.29–2.82]), amcenestrant (OR 1.96 [95% CI, 0.63–3.28]) and sacituzumab govitecan (OR 2.00 [95% CI, 0.71–3.29]) had a higher number of grade ≥ 3 non-hematological toxicities compared to ribociclib; however, this was not statistically significant. For two agents, xentuzumab and venetoclax, a lower number of grade ≥ 3 non-hematological AEs were documented, but their 95% CI also crossed 1 (Supplemental Figure S5b). This analysis is limited by the difference in control arms, which were ET for most studies but chemotherapy for some [17,18,29,32].

## 4. Discussion

In this study, we performed a systematic review of phase-II and -III randomized trials in ER + MBC after progression on a CDK4/6i. Treatment decisions in this space can be informed by the presence of molecular alterations [11]. In an attempt to make meaningful comparisons that could potentially assist clinical decisions, and recognizing the variability in this disease with different molecular alterations and their targeted therapy, we grouped our analysis into drugs with similar mechanisms of action.

In this network meta-analysis of treatment after progression on a CDK4/6i in patients with ER + MBC, we found that in HER2-low tumors, trastuzumab deruxtecan and sacituzumab govitecan showed similar efficacy (PFS and OS), with higher toxicity for sacituzumab govitecan, driven by neutropenia and diarrhea. For patients with AKT pathway-mutated tumors, capivasertib showed non-significantly improved efficacy and fewer side effects compared to alpelisib. Very few trials reported OS results; therefore, our results were based mainly on PFS and adverse effects.

For patients with ER + MBC, CDK4/6i with ET, either aromatase inhibitors or fulvestrant, is the standard first-line therapy of choice in most patients [1,2,11,13]. Almost all patients eventually develop resistance to these therapies, with a median PFS of about two years in the first-line setting [3,5,6]. Although several trials have tried to address the question of the optimal treatment strategy post-progression on a CDK4/6i, management decisions are often based on observational data or cross-trial comparisons due to a lack of direct efficacy comparisons [12,13].

Subgroup analysis for HER2-low tumors showed similar efficacy for sacituzumab govitecan and trastuzumab deruxtecan in our analysis. While trastuzumab deruxtecan was evaluated in the large randomized DESTINY-Breast 04 trial, specifically in patients with HER2-low tumors, data for sacituzumab govitecan were derived from an exploratory analysis of TROPICS-02 [17,35]. Patients in TROPICS-02 were more heavily treated than those in DESTINY-Breast 04, which could have led to the relative underperformance of Sacituzumab govitecan in this setting. Although no patients in the TROPICS-02 trial received T-DXd prior to receiving sacituzumab govitecan, data from multiple real-world studies suggest very modest efficacy for sequencing ADCs [42,43,44]. Once the results of ASCENT-7 (sacituzumab govitecan vs. physician choice chemotherapy in ER + MBC after progression on CDK4/6i) become available, trastuzumab deruxtecan may be the preferred option in HER2-low disease [45]. In contrast, sacituzumab govitecan remains the only ADC option in patients with no HER2 expression on their tumors [46]. High toxicity seen with sacituzumab govitecan seems to be driven by higher rates of neutropenia and diarrhea. Clinical practice and trial analysis affirm that about (35–50)% of patients require growth factor support with sacituzumab govitecan [47,48]. Reassuringly, the rate of febrile neutropenia in the TROPICS-02 trial was 5%, with one death secondary to neutropenic colitis [39]. On the other hand, pneumonitis remains the major toxicity concern with trastuzumab deruxtecan (10–15% risk of all grade pneumonitis and <1% risk of death) [49].

Both trastuzumab deruxtecan and sacituzumab govitecan are ADCs with large phase-III trials showing efficacy in HER2-low ER + MBC and heavily pre-treated ER + MBC irrespective of HER2 status [17,39]. The data for trastuzumab deruxtecan and sacituzumab govitecan need to be interpreted in the knowledge that the pivotal phase-III trials that led to their approvals in ER + MBC (DESTINY breast 04 and TROPICS-02) included heavily pre-treated patients (median of three lines of treatment in DESTINY-Breast 04 and four lines of chemotherapy in TROPICS-02 apart from prior ET). Hence, these data may not be directly applicable to the second-line setting, and we recommend following approval guidelines for the use of these agents until further data are available.

Subgroup analysis for PI3K pathway-mutated tumors showed better efficacy and superior safety for capivasertib than for alpelisib for PI3K pathway-altered tumors; however, statistical significance was not achieved. Additionally, it remains uncertain whether the benefit of capivasertib is restricted to patients with AKT pathway-altered tumors, or whether patients with no alterations in the AKT pathway benefit as well. The FDA has approved capivasertib only for patients with an AKT pathway alteration based on a subgroup analysis from the Capitello-291 study showing no significant benefit in patients without an AKT pathway mutation [15,50]. Comparing the toxicity between different PI3K/AKT/mTOR inhibitors, capivasertib seems to be less toxic. Although there seems to be a higher propensity for diarrhea, mucositis and hyperglycemia are less frequent. In the toxicity analysis from the Capitello-291 trial [15], only 2% had grade 3 hyperglycemia compared to 37% with alpelisib in the SOLAR-1 trial [14]. Given the detrimental effect on quality of life with alpelisib and no statistically significant OS advantage even in PIK3CA-mutated tumors [14,37] we await the final quality of life and OS data from Capitello-291, so that they can inform therapeutic decisions.

Elacestrant is an oral SERD approved for patients with an ESR1 mutation detectable by companion liquid biopsy [51]. However, in our analysis, although there were some benefits to elacestrant compared to ET alone, it performed worse than continuing ribociclib, with an HR of 1.57 in patients with ESR1 mutation. This corresponds to the relatively modest benefits seen with elacestrant in the EMERALD trial overall, along with the caveats of the subpar control arm [11,16]. Its relative lack of efficacy, even in ESR1-mutant tumors, raises questions regarding its efficacy and points to the need for a better, more sensitive biomarker to better identify tumors that may respond.

In an exploratory analysis, we compared all treatments with the strategy used in the MAINTAIN trial [19], given the modest improvements that have been demonstrated with continuing CDK4/6i beyond CDK4/6i progression [19,20,52], and hypothesized that effective treatments should be able to outperform ribociclib in this setting. We found that no drug showed unequivocal superiority over the switching/continuation of a CDK4/6i with a change in ET. There was a trend towards benefits for capivasertib and Trastuzumab deruxtecan. While this study was being reviewed for publication, the results of the postMONARCH [53] trial, which evaluated the use of abemaciclib and fulvestrant after progression on a CDK4/6i +ET, were published. Similarly to the results of the MAINTAIN study, the abemaciclib strategy demonstrated a benefit in terms of PFS (HR, 0.73; 95% CI, 0.57–0.95). The majority of the patients (60% [53] and 86% [19]) in both these studies received palbociclib as first-line CDK4/6i. We could not compare overall survival among different treatments in this setting to ribociclib, as there were no OS data for the trial by Kalinsky et al. [19], and moreover, only three trials reported OS data with HR for CDK 4/6i-exposed patients. This is a part of the growing tendency among industry-sponsored studies to design trials with the primary endpoint of PFS in a setting where the prognosis is limited and the OS endpoint is easily achievable. The previous literature consistently shows a lack of surrogacy between PFS and OS in metastatic ER+ breast cancer [54], and a more thoughtful trial design with more patient-centric endpoints is encouraged [55,56]. In the absence of OS data, benefits in terms of PFS need to be understood in the context of the implications of adverse effects and quality of life, which require individualized discussions with the patients regarding their care [57]. The tradeoffs between the specific toxicities of each agent and their impact on quality of life could play an important role in therapeutic decisions if efficacy data appear equivocal, with no clear winner.

Our study had limitations. First, the data used for the network meta-analysis were derived from trials performed in different lines of treatment for ER + MBC, leading to heterogeneity. This has been discussed in detail while interpreting the results. Moreover, OS data were not available for several of the included studies, and most recommendations are based on PFS analysis, which needs to be considered while interpretating the results of the study. We did not perform a quality of life analysis, expecting these data to be highly heterogenous, which may have aided in further understanding our results. Second, our analysis was based on summary statistics rather than individual patient data. This may introduce uncertainty in the analysis, particularly as, in the absence of individual participant data, sensitivity analyses based on molecular subgroups are limited. However, individual patient data were inaccessible for these trials as many are still ongoing.

## 5. Conclusions

In conclusion, the management of ER + MBC after progression on a CDK4/6i and ET remains a significant clinical challenge, with modest activity shown by various systemic therapy agents. Given the molecular heterogeneity of this disease, deciding on the next best line of therapy requires categorization into a molecular subgroup. Both ADCs (sacituzumab govitecan and Trastuzumab deruxtecan) seem to have similar activity in this space for HER2-low, and ongoing clinical trials will clarify their place in the management algorithm for ER + MBC. Given their similar efficacy and different toxicity profiles, Trastuzumab deruxtecan takes priority for HER2-low tumors, whereas sacituzumab govitecan may be preferred for patients with chemo-refractory HER2 non-expressing tumors. Capivasertib is promising, with a manageable safety profile, especially compared to alpelisib. Several targeted agents have been developed and tested in this space, including several oral SERDs; however, their efficacy is modest at best and does not seem better than continuing/switching to another CDK4/6i.

Although data from several trials are available, few trials have been designed with OS as a primary endpoint, and PFS results are variable, making decision-making challenging.

## Figures and Tables

**Table 1:** Study design and outcomes in the included studies.

No	Study	Trial Design	Population	Line of Treatment	Treatment Arms	N	Primary Outcome Measure	Relevant Secondary Outcomes
1	Rugo 2022 [39] (TROPiCS 02)	III	ER+/HER2–MBC post 2–4 prior systemic CT including one CDK4/6i, one taxane, and ET	Median 3 (range, 0–8) lines of CT in MBC	Sacituzumab govitecan vs. TPC (TPC: Eribulin Cape Gem VBL)	272 vs. 271	PFS	OS, ORR, CBR, QoL, AE
2	Lindeman 2022 [36] (VERONICA)	II	ER+/HER2– MBC after ≤2 lines of prior ET including a CDK 4/6i; prior chemotherapy, SERD, BCL2 inhibitors not allowed	Up to 2 lines of ET, no chemo, 80% had 1 line of treatment	Venetoclax + fulvestrant vs. fulvestrant	51 vs. 52	CBR	PFS, OS, and AE
3	Bidard 2022 [16] (EMERALD)	III	ER+ HER2-MBC, after ≤2 lines of ET, all PD on CDK 4/6i and fulvestrant/AI, 1 line of chemotherapy allowed	43.4% received 2 lines ET. 22.2% received 1 line of chemo; the rest received no chemo	Elacestrant vs. SOC fulvestrant, anastrozole, letrozole, or exemestane monotherapy	239 vs. 238	PFS in all pts and those with ESR1 mutation	OS in all pts and in those with ESR1 mutation, PFS and OS in those without ESR1 mutation, ORR, CBR, AE
4	Andre 2019 ^#^ [14] (SOLAR 1)	III	ER + HER2-MBC after first-line ET CT for MBC, fulvestrant, no PI3K, AKT, or mTOR inhibitor allowed	Second line	Alpelisib + fulvestrant vs. placebo + fulvestrant	169 vs. 172	PFS in mPI3KCA pts	OS in mPI3KCA pts, ORR, CBR, AE, PFS, and OS in pts without PI3KCA mutation
5	Dent 2021 [28] (SANDPIPER)	III	ER + HER2-MBC with mPI3KCA after 1 line of ET Prior PI3K/AKT/mTOR inhibitor not allowed	Median 1 line prior	Taselisib + fulvestrant vs. placebo + fulvestrant	340 vs. 176	PFS	OS, ORR, CBR, QoL, AE
6	Turner 2021 [32] (Ipatunity 130, cohort B)	III	ER+ HER2-MBC with PIK3CA/AKT1/PTEN alteration	Pts had (neo)adjuvant CT—55%; ET MBC—46%; PI3K/ mTOR—24%; and CDK4/6i—26%	Ipatasertib + paclitaxel vs. placebo + paclitaxel	146 vs. 76	PFS	OS, ORR, CBR, AE, PRO
7	García-Sáenz 2022 [31] Only PFS data as subgroup, no OS or ORR available	II, 3 arm	ER + HER2- MBC PD on AI, 1 line of chemotherapy allowed Prior PI3K/AKT/mTOR inhibitor not allowed	1 line prior	Sapanisertib 4 mg OD + Fulvestrant vs. Sapanisertib 30 mg weekly + fulvestrant vs. Fulvestrant	47 vs. 47 vs. 46	PFS	OS, ORR, CBR, QoL, AE
8	Modi 2022 * [17] (DESTINY BREAST 04)	III	HER2-low MBC in 2nd line and beyond	Median 3 lines	Trastuzumab deruxtecan vs. TPC (capecitabine, eribulin, gemcitabine, paclitaxel, or nab-paclitaxel)	331 vs. 163	PFS in HR+ MBC	PFS in all pts, OS in ER + MBC, OS in all pts, ORR < AE
9	O’Shaughnessy 2021 * [29]	II	ER + MBC with Ki 67 >15% or grade 3 TNBC, taxane-naïve ≥12 m prior to advanced disease setting, 20% of ER+ received CDK4/6i	30% had one line of CT in MBC setting, 60% in curative	Paclitaxel + alisertib vs. Paclitaxel alone	70 vs. 69	PFS	ORR, CBR, AE, OS
10	Conolly 2021 [30] (E2112 ECOG ACRIN)	III	ER+ HER2- MBC, PD post AI, Fulvestrant, CDK 4/6i and 1 line of CT permitted	Median 3 lines of treatment	Entinostat + exemestane v placebo + exemestane	305 vs. 303 (35%)	PFS and OS (co-primary)	AE, TTD, ORR, PROs
11	Kalinsky 2022 [19] (MAINTAIN)	II	ER+ HER2- MBC with PD on any CDK4/6i	palbociclib (84%), ribociclib (11%), abemaciclib (2%), and palbociclib and another CDK 4/6i (3%)	ET (fulvestrant or exemestane) + Ribociclib vs. ET + placebo	60 vs. 59	PFS	AE, ORR
12	Tolaney 2022 [40] (AMEERA-3)	II	ER+ HER2-MBC for 2L/3L tt, CDK 4/6i allowed, 1 line CT and ≤2 lines ET	Up to 2 lines of prior therapy, 79% post CDK 4/6i	Amcenestrant vs. ET (fulvestrant/tamoxifen/AI)	143 vs. 147	PFS	OS, AE
13	Turner 2023 [15] (CAPITELLO 291)	III	ER + HER2-MBC, CDK4/6i allowed	-	Capivasertib + fulvestrant vs. placebo + fulvestrant.	355 vs. 353	PFS in overall and AKT mutate population	OS, ORR, CBR, AE
14	Schimd 2023 [41] (XENERA-1)	II	ER + HER2-MBC, CDK4/6i allowed	-	xentuzumab (IGF1/2 Mab) + everolimus + exemestane vs. everolimus + exemestane	52 vs. 51	PFS	OS, DCR, ORR, time to pain progression

Abbreviations: AE, adverse event; AI, aromatase inhibitor; AKT, protein kinase B; Cape, capecitabine; CBR, clinical benefit rate; CDK 4/6i, cyclin-dependent kinase 4/6 inhibitor; CT, chemotherapy; ER+, endocrine (estrogen/progesterone) receptor-positive; ESR1, estrogen receptor 1; ET, endocrine therapy; HER2-, Human epidermal growth factor receptor 2-negative; MBC, metastatic breast cancer; ORR, overall response rate; OS, overall survival; PD; progression; PI3KCA, phosphatidylinositol-4,5-bisphosphate 3-kinase catalytic subunit alpha; PFS, progression-free survival; PROs, patient-reported outcomes; PTEN, Phosphatase and TENsin homolog deleted on chromosome 10; QoL, quality of life; RCT, randomized controlled trial; TPC therapy of physician’s choice; vs., versus. ^#^ mPI3KCA cohort shown. * Only ER + MBC data shown.

**Table 2:** Characteristics of the studies included.

Study ID	Intervention/Control	Duration of Response	Age (yr)	Menopausal Status	Visceral Mets (%)	Liver Mets (%)	De Novo MBC (%)	Median Lines of Prior Treatment	Prior ET	Prior CT	CDK 4/6i-Exposed	Duration of CDK 4/6i	Endocrine Resistance
Rugo 2022 [35,39] (TROPiCS 02)	Sacituzumab govitecan vs. TPC	7.4 m vs. 5.6 m	57 55	-	95%	84%	29%	7	-	Median 4 lines	98%	40% on CDK 4/6i ≥ 12 m	Entire population defined as ‘endocrine resistant’
Lindeman 2022 [36] (VERONICA)	Venetoclax + fulvestrant vs. fulvestrant	-	60 58	Pre-/post	87%	61%	-	1	80% had 1 line of ET	none	100%	15 m vs. 16.5 m	43% had ESR1 and 41% had TP53 mutations
Bidard 2022 [16] (EMERALD)	Elacestrant vs. ET (fulvestrant/AI)	Not calculable vs. 5.55 m	63 64	post	70%	-	-	1	43% had 2 prior lines	22% had 1 line of CT	100%	-	48.7% had the ESR1 mutation
Andre 2019 ** [14,37] (SOLAR 1)	Alpelisib + fulvestrant vs. placebo + fulvestrant	-	63 64	post	57%	30%	-	1	Fulvestrant not allowed	61% exposed to CT	5.9%	-	13% primary resistance ^#^, 72% secondary resistance
Dent 2021 [28] (SANDPIPER)	Taselisib + fulvestrant vs. placebo + fulvestrant	8.7 m vs. 7.2 m	60 61	post	89%	-	-	1	Fulvestrant not allowed	31% exposed to CT	2.9%	-	26% endocrine-resistant
Turner 2021 *** [32] (Ipatunity 130, cohort B)	Ipatasertib + paclitaxel vs. placebo + paclitaxel	9.2 m vs. 9.2 m	58 56	78% post	80%	51%	97%	-	46% had at least 1 line of ET	55% exposed to CT	26%	-	18% primary resistance and 45% secondary
García-Sáenz 2022 [31]	Sapanisertib OD + fulvestrant vs. Sapanisertib wk + fulvestrant vs. Fulvestrant	-	59 57 60	post	65%	-	-	1	-	Up to 1 line of CT allowed	34%	-	16% endocrine-resistant
Modi 2022 * [17] (DESTINY BREAST 04)	Trastuzumab deruxtecan vs. TPC	10.7 m vs. 6.8 m	56.8 55.7	Pre- and post	90%	73%	-	3	-	100% exposed to CT	70.4%	-	Patients included were ‘endocrine refractory’; i.e., they progressed on 1 line of ET, and the investigator believed that they would no longer benefit from further treatment with ET
O’Shaughnessy 2021 * [29]	Alisertib + paclitaxel vs. paclitaxel	-	62.5 63.2	post	96%	54%	-	-	90% AI, 32% tamoxifen, 29% fulvestrant	30% CT in metastatic setting, 41% prior taxane in curative setting	20.1%	-	-
Conolly 2021 [30] (E2112 ECOG ACRIN)	Entinostat + exemestane vs. Placebo + exemestane	-	63 63	95% post	60%	-	-	1	30% exposed to fulvestrant	60% prior CT	35%	-	84% resistant to AI
Kalinsky 2022 [19] (MAINTAIN)	Ribociclib + ET vs. Placebo + ET	-	55 59		60%	-	45%	1	18% post 2 lines of ET	9.2% exposed to CT	100%	15.5 m vs. 17 m	42.3% of those who received fulvestrant as ET had the ESR1 mutation. Co-occurring alterations: TP53 mutations (30.3%), CCND1 alterations (24.2%), PIK3CA mutations (21.2%), and FGFR1 alterations (18.2%).
Tolaney 2022 [40] (AMEERA-3)	Amcenestrant vs. ET	-	58 60	84% post	64%	-	-	1	93.4% exposed to ET, 9.7% to SERD	11.4% exposed to CT	78.9%	-	41.4% ESR1-mutated, 4.8% primary endocrine resistance, 94.8% secondary
Turner 2023 ^$^ [15] (CAPITELLO 291)	Capivasertib + fulvestrant vs. placebo + fulvestrant	-	59 58	80% post	68%	43.2%	-	1	76% received at least 1 line of ET; 11% received at least 2 lines of ET	68% exposed to CT	69%	-	37% primary and 63% secondary resistance
Schimd 2023 [41] (XENERA-1)	Xentuzumab + everolimus + exemestane vs. placebo + everolimus + exemestane		60.5 59	94% post	31.1% did not have bone only disease; the rest did	-	-	-	43.7% fulvestrant	8.7% exposed to CT	75.7%	-	26.2% primary and 73.8% secondary resistance

Abbreviations: AKT, protein kinase B; AI, aromatase inhibitor; CCDN1, cyclin D1; CT, chemotherapy; ESR1, estrogen receptor 1; ET, endocrine therapy; FGFR1, Fibroblast Growth Factor Receptor 1; m, months; met, metastasis; N, sample size; OD, once daily; PI3KCA, Phosphatidylinositol 3-kinase; PTEN, Phosphatase and tensin homolog; SERD, selective estrogen receptor down-regulator; TPC, treatment of physician’s choice (chemotherapy); wk, weekly; yrs, years. * Estrogen receptor-positive cohort only shown. ^$^ 40.8% had AKT pathway alterations. ^#^ Prmary resistance defined as relapse within 24 months while the patient was receiving adjuvant endocrine therapy or progression within 6 months while the patient was receiving endocrine therapy for advanced disease, all others considered secondary resistance. Endocrine sensitive disease excluded. ** Only PI3KCA-mutated cohort shown. *** All are PIK3CA/AKT1/PTEN-altered.

**Table 3:** Results of the studies included.

No.	Study	OS [HR, (95% CI)]	OS (Median)	PFS [HR, (95% CI)]	PFS (Median)	Grade ≥ 3 AE
1.	Rugo 2022 [35,39] (TROPiCS 02)	0.79 (0.65–0.96), *p* 0.02	14.4 m vs. 11.2 m	0.66 (0.53–0.83), *p* 0.003 HER2-low population: 0.58 (0.42–0.79)	5.5 m vs. 4.0 m	Neutropenia (51% vs. 38%), lymphopenia (9% vs. 5%), anemia (6% vs. 3%), diarrhea (9% vs. 1%), febrile neutropenia 5% vs. 4%) and fatigue (6% vs. 2%)
2.	Lindeman 2022 [36] (VERONICA)	1.87 (1.02- 3.43), *p* 0.0403	19.7 m vs. NE	0.94 (0.61–1.45), *p* 0.7853	2.69 m vs. 1.94 m	All (26% vs. 11.8%), fatigue (6% vs. 2%), neutropenia (12% vs. 0%), lymphopenia (4% vs. 0%) dyspnea (4% vs. 0%)
3.	Bidard 2022 [16] (EMERALD)	0.75 (0.54–1.04), *p* 0.08 In ESR1-mutated: 0.59 (0.36–0.96), *p* 0.03	-	0.70 (0.55–0.88), *p* 0.002 In ESR1-mutated: 0.55 (0.39 to 0.77), *p* 0.0005	2.8 m vs. 1.9 m In ESR1-mutated: 3.8 m vs. 1.9 m	All (27% vs. 20.5%), nausea (2.5% vs. 0.9%), back pain (2.5% vs. 0.9%), increased ALT (2.1% vs. 0.9%)
4.	Andre 2019 [14,37] (SOLAR 1) *PI3KCA-mutated cohort	0.86 (0.64–1.15), *p* 0.15	39.3 m vs. 31.4 m	0.65 (0.50–0.85), *p* < 0.001 For CDK 4/6i-exposed patients: 0.48 (0.17–1.36)	11 m vs. 5.7 m	Hyperglycemia (36.6% vs. 0.7%) rash (9.9% vs. 0.3%), maculopapular rash (8.8% vs. 0.3%), diarrhea (6.7% vs. 0.3%)
5.	Dent 2021 [28] (SANDPIPER)	NO HR, 73/340 vs. 43/176 events, immature	-	0.70 (0.56–0.89) *p* 0.0037 In CDK 4/6i-exposed: 0.62 (0.15–2.49)	7.4 m vs. 5.4 m In CDK 4/6i-exposed: 6.1 m vs. 5.6 m	Overall (49.5% vs. 16.4%), diarrhea (11.5% vs. 0.9%), hyperglycemia (10.8% vs. 0.5%), rash (3.8% vs. nil), stomatitis (3.6% vs. nil), colitis (3.1% vs. nil), hypertension (2.4% vs. 3.3%)
6.	Turner 2021 [32] (Ipatunity 130, cohort B)	0.72 (0.42–1.24), immature	NE vs. 20.9 m	1.00 (0.71–1.40), *p* 0.997 In CDK 4/6i-exposed: 0.85 (0.45–1.60)	9.3 m vs. 9.3 m In CDK 4/6i-exposed: 7.3 m vs. 5.6 m	Overall (55% vs. 47%), diarrhea (12% vs. 1%), neutrophil count decreased (9% vs. 7%), neutropenia (8% vs. 9%), peripheral neuropathy (7% vs. 3%), peripheral sensory neuropathy (3% vs. 5%), and hypertension (1% vs. 5%)
7.	García-Sáenz 2022 [31] (Three-arm study)	OD ^#^: 0.71 (0.36–1.40), *p* 0.276 weekly ^#^: 0.89 (0.47–1.68), *p* 0.470	NE vs. 30.5 m 34.2 m vs. 30.5 m	OD ^#^: 0.77 (0.47–1.26), *p* 0.537 weekly ^#^: 0.88 (0.53–1.45), *p* 0.849 In CDK 4/6i-exposed: OD ^#^: 0.34 (0.14–0.82), *p* 0.042 weekly ^#^: 0.48 (0.21–1.12), *p* 0.116	7.2 m vs. 3.5 m 5.6 m vs. 3.5 m	Grade ≥ 3 in 68.1% (OD) vs. 53.2%(weekly) vs. 0% (control). Nausea (0 vs. 8.5%), vomiting (4.3% vs. 14.9%), diarrhea (10.6% vs. 4.3%), stomatitis (8.5% vs. 4.3%), fatigue (2.1% vs. 10.6%), asthenia (8.5% vs. 6.4%), hyperglycemia (6.4% vs. 2.1%), rash (14.9% vs. 2.1%), pruritic (4.3% vs. 2.1%), weight loss (4.3% vs. 2.1%), increased GGT (6.4% vs. 2.1%)
8.	Modi 2022 [17] (DESTINY BREAST 04)	0.64 (0.48–0.86), *p* 0.003	23.9 m vs. 17.5 m	0.55 (0.42–0.73), *p* < 0.001 In CDK 4/6i-exposed: 0.55 (0.42–0.73)	10.1 m vs. 5.4 m In CDK 4/6i-exposed: 10.0 m vs. 5.4 m	Gr ≥ 3 AEs in 52.6% vs. 67.4%: neutropenia (13.7% vs. 40.7%), anemia (8.1% vs. 4.7%), thrombocytopenia (5.1% vs. 0.6%), leucopenia (6.5% vs. 19.2%), nausea (4.6% vs. 0), increased ALT (3.2% vs. 8.1%), fatigue (7.5% vs. 4.7%), loss of appetite (2.4% vs. 1.2%)
9.	O’Shaughnessy 2021 [29] .	0.89 (0.58–1.38), *p* 0.61	26.3 m vs. 25.1 m	0.56 (0.37–0.84), *p* 0.005 In CDK 4/6i-exposed: 0.58 (0.26–1.32), *p* 0.19	10.2 m vs. 7.1 m In CDK 4/6i-exposed: 13.9 m vs. 5.6 m	Neutropenia (59.5% vs. 16.4%), anemia (9.5% vs. 1.2%), diarrhea (10.7% vs. 0), stomatitis (15.5% vs. 0), and neuropathy (1.5% vs. 11.4%)
10.	Conolly 2021 [30] (E2112 ECOG ACRIN)	0.99 (0.82–1.21), *p* 0.94.	23.4 m vs. 21.7 m	0.87 (0.67–1.13), *p* 0.30	3.3 m vs. 3.1 m	Entinostat-related grade ≥ 3 AEs: neutropenia (20%), hypophosphatemia (14%), anemia (8%), leukopenia (6%), fatigue (4%), and diarrhea (4%), Gr ≥ 3 AE overall: Entinostat vs. placebo: 51% vs. 16%, *p* < 0.001
11.	Kalinsky 2022 [19] (MAINTAIN)	No HR, 20% vs. 11% *p* 0.51	-	0.56 (0.37–0.83), *p* 0.004	5.33 m vs. 2.76 m	Grade ≥ 3 neutropenia 40% vs. 1.6%, anemia 1.7% in each, 3.3% vs. none for febrile neutropenia, QTc prolongation 1.7% vs. none, pneumonitis 1.7% vs. none
12.	Tolaney 2022 [40] (AMEERA-3)	0.913 (0.60–1.4) immature	-	1.051 (0.789–1.4), *p* 0.6437 ESR1-mutated: 0.9 (0.57–1.44)	3.6 m vs. 3.7 m ESR1mutated: 3.7 m vs. 2 m	21.7% vs. 15.6% of pts had grade ≥ 3 TRAEs, back pain 1.4% vs. 0.7%, nausea 2.1% vs. none, fatigue 1.4% vs. none
13.	Turner 2023 [15] (CAPITELLO 291)	0.74 (0.56–0.98) AKT-altered: 0.69 (0.45–1.05)	-	0.60 (0.51–0.71), *p* < 0.001 AKT-altered: 0.50 (0.38–0.65), *p* < 0.001	7.2 m vs. 3.6 m AKT-altered: 7.3 m vs. 3.1 m	Gr ≥ 3 AE rash (12.1% vs. 0.3%), diarrhea (9.3% vs. 0.3%), and hyperglycemia (2.3% vs. 0.3%); grade ≥ 3 stomatitis (2.0% vs. 0%)
14.	Schimd 2023 [41] (XENERA-1)	No HR, 9.6% vs. 15.7% died, immature	-	1.19 (0.55–2.59), *p* 0.6534	12.7 m vs. 11 m	Grade ≥ 3 overall 56% vs. 54.9%, diarrhea 6% vs. none, nausea 4% vs. none, fatigue 8% vs. 1%, hyperglycemia 2% vs. 5.9%

Abbreviations: AE, adverse event; AKT, protein kinase B; ALT, alanine transaminase; CDK4/6i, cyclin-dependent kinase 4/6 inhibitor; CI, confidence interval; ESR1, Estrogen Receptor 1; GGT, gamma glutamyltransferase; HER2, human epidermal growth factor receptor 2; HR, hazard ratio; m, months; NE, not evaluable; OD, once daily; OS, overall survival; PFS, progression-free survival; TRAEs, treatment-related adverse events; vs., versus. ^#^: sapanisertib was given in two doses in this three-arm study, once daily (OD) or once every week (weekly).

## Data Availability

Data are available on request; contact Dr Atul Batra, batraatul85@gmail.com.

## Supplementary Materials

The following supporting information can be downloaded at: https://www.mdpi.com/article/10.3390/curroncol32010053/s1, Figure S1a, Traffic plot for risk of bias assessment; Figure S1b, Summary plot for risk of bias assessment; Figure S2, Forest plot showing the network meta-analysis of progression-free survival through the network meta-analysis of oral selective estrogen receptor degraders to continuing ribociclib in ESR1-mutated patients; Figure S3, Forest plot showing network meta-analysis of adverse events of grade 3 and above for selective estrogen receptor degraders; Figure S4, Forest plot showing network meta-analysis of progression-free survival for all interventions compared to that for continuing ribociclib; Figure S5a, Forest plot of network meta-analysis comparison of grade ≥ 3 hematological toxicities vs. ribociclib; Figure S5b, Forest plot of comparison network meta-analysis of non-hematological grade ≥ 3 adverse effects of all agents vs. ribociclib, and Supplemental File S1 with search strategy and supplementary tables: Supplement_file_NM. References [58,59,60,61,62,63,64,65,66,67,68,69,70,71,72,73,74,75,76,77,78,79,80,81,82,83,84,85,86,87,88,89,90,91,92,93] can be found in the supplementary file.
