# Supplementary material for: Systematic Review and Network Meta-Analysis on Treating Hormone Receptor-Positive Metastatic Breast Cancer After CDK4/6 Inhibitors
Source: Curr Oncol. 2025 Jan 20;32(1):53. doi: 10.3390/curroncol32010053 (PMC11763720; doi:10.3390/curroncol32010053)
Supplement: Supplementary file 1 [file curroncol-32-00053-s001.zip › Supplement_file_NM.pdf]

Supplement Section S1: summary and pictorial representation of our search strategy across databases

S1.1.MEDLINE

Search number	Query	Sort By	Filters	Search Details	Results	Time
19	((((((((breast cancer*[MeSH Terms]) OR (breast tumor*[MeSH Terms])) OR (breast neoplasm[MeSH Terms])) OR (breast carcinoma[MeSH Terms])) OR (breast tumour[MeSH Terms])) AND ((((metastatic[Text Word]) OR (palliative[Text Word])) OR (advance*[Text Word])) OR (relapse*[Text Word]))) AND ((((((((((hormone receptor[Text Word]) OR (hormone receptor positive[Text Word])) OR (endocrine receptor positive[Text Word])) OR (endocrine receptor[Text Word])) OR (estrogen receptor positive[Text Word])) OR (estrogen receptor[Text Word])) OR (ER positive[Text Word])) OR (progesterone receptor[Text Word])) OR (progesterone receptor positive[Text Word])) OR (PR positive[Text Word])))) AND ((((((((drug therapy[MeSH Terms]) OR (immunotherapy[MeSH Terms])) OR ((((((((((((citrate, tamoxifen[MeSH Terms]) OR (anastrozole[MeSH Terms])) OR (letrozole[MeSH Terms])) OR (exemestane[Supplementary Concept])) OR (toremifene[MeSH Terms])) OR (endocrine therapy[Text Word])) OR (hormone therapy[Text Word])) OR (selective estrogen receptor modulator[MeSH Terms])) OR (SERD[Text Word])) OR (SERM[Text Word])) OR (selective estrogen receptor downregulator[Text Word])) OR (fulvestrant[MeSH Terms]))) OR (((Phosphoinositide-3 Kinase Inhibitors[MeSH Terms]) OR (Alpelisib[Supplementary Concept])) OR (Taselisib[Supplementary Concept]))) OR ((MTOR Inhibitors[MeSH Terms]) OR (everolimus[MeSH Terms]))) OR ((Angiogenesis Inhibitors[MeSH Terms]) OR (Bevacizumab[MeSH Terms]))) OR (Antineoplastic	Publication Date	Randomized Controlled Trial	(("breast cancer*"[MeSH Terms] OR (("breast"[MeSH Terms] OR "breast"[All Fields] OR "breasts"[All Fields] OR "breast s"[All Fields]) AND "tumor*"[MeSH Terms]) OR "breast neoplasms"[MeSH Terms] OR "breast neoplasms"[MeSH Terms] OR "breast neoplasms"[MeSH Terms]) AND ("metastatic"[Text Word] OR "palliative"[Text Word] OR "advance*"[Text Word] OR "relapse*"[Text Word]) AND ("hormone receptor"[Text Word] OR "hormone receptor positive"[Text Word] OR "endocrine receptor positive"[Text Word] OR "endocrine receptor"[Text Word] OR "estrogen receptor positive"[Text Word] OR "estrogen receptor"[Text Word] OR "er positive"[Text Word] OR "progesterone receptor"[Text Word] OR "progesterone receptor positive"[Text Word] OR "pr positive"[Text Word]) AND ("drug therapy"[MeSH Terms] OR "immunotherapy"[MeSH Terms] OR ("tamoxifen"[MeSH Terms] OR "anastrozole"[MeSH Terms] OR "letrozole"[MeSH Terms] OR "exemestane"[Supplementary Concept] OR "toremifene"[MeSH Terms] OR "endocrine therapy"[Text Word] OR "hormone therapy"[Text Word] OR "selective estrogen receptor modulators"[MeSH Terms] OR "SERD"[Text Word] OR "SERM"[Text Word] OR "selective estrogen receptor downregulator"[Text Word] OR "fulvestrant"[MeSH Terms]) OR ("phosphoinositide 3 kinase inhibitors"[MeSH Terms] OR "alpelisib"[Supplementary Concept] OR "2 3 2 1 isopropyl 3 methyl 1h 1 2 4 triazol 5 yl 5 6 dihydrobenzo f imidazo 1 2 d 1 4 oxazepin 9 yl 1h pyrazol 1 yl 2 methylpropanamide"[Supplementary Concept]) OR ("mtor inhibitors"[MeSH Terms] OR "everolimus"[MeSH Terms]) OR ("angiogenesis inhibitors"[MeSH Terms] OR "bevacizumab"[MeSH Terms]) OR "antineoplastic agents"[MeSH Terms] OR ("cyclin dependent kinase inhibitor proteins"[MeSH Terms] OR "palbociclib"[Supplementary Concept] OR "abemaciclib"[Supplementary Concept] OR "ribociclib"[Supplementary	626	09:58:14

	Agents[MeSH Terms])) OR ((((Cyclin-Dependent Kinase Inhibitor Proteins[MeSH Terms]) OR (palbociclib[Supplementary Concept])) OR (abemaciclib[Supplementary Concept])) OR (ribociclib[Supplementary Concept])))			Concept]))) AND (randomizedcontrolledtrial[Filter])		
**18**	((((((((breast cancer*[MeSH Terms]) OR (breast tumor*[MeSH Terms])) OR (breast neoplasm[MeSH Terms])) OR (breast carcinoma[MeSH Terms])) OR (breast tumour[MeSH Terms])) AND ((((metastatic[Text Word]) OR (palliative[Text Word])) OR (advance*[Text Word])) OR (relapse*[Text Word]))) AND ((((((((((hormone receptor[Text Word]) OR (hormone receptor positive[Text Word])) OR (endocrine receptor positive[Text Word])) OR (endocrine receptor[Text Word])) OR (estrogen receptor positive[Text Word])) OR (estrogen receptor[Text Word])) OR (ER positive[Text Word])) OR (progesterone receptor[Text Word])) OR (progesterone receptor positive[Text Word])) OR (PR positive[Text Word])))) AND ((((((((drug therapy[MeSH Terms]) OR (immunotherapy[MeSH Terms])) OR ((((((((((((citrate, tamoxifen[MeSH Terms]) OR (anastrozole[MeSH Terms])) OR (letrozole[MeSH Terms])) OR (exemestane[Supplementary Concept])) OR (toremifene[MeSH Terms])) OR (endocrine therapy[Text Word])) OR (hormone therapy[Text Word])) OR (selective estrogen receptor modulator[MeSH Terms])) OR (SERD[Text Word])) OR (SERM[Text Word])) OR (selective estrogen receptor downregulator[Text Word])) OR (fulvestrant[MeSH Terms]))) OR (((Phosphoinositide-3 Kinase Inhibitors[MeSH Terms]) OR (Alpelisib[Supplementary Concept])) OR (Taselisib[Supplementary Concept]))) OR ((MTOR Inhibitors[MeSH Terms]) OR (everolimus[MeSH Terms]))) OR ((Angiogenesis Inhibitors[MeSH Terms]) OR (Bevacizumab[MeSH Terms]))) OR (Antineoplastic Agents[MeSH Terms])) OR ((((Cyclin-Dependent Kinase Inhibitor Proteins[MeSH Terms]) OR (palbociclib[Supplementary	Publication Date		("breast cancer*"[MeSH Terms] OR (("breast"[MeSH Terms] OR "breast"[All Fields] OR "breasts"[All Fields] OR "breast s"[All Fields]) AND "tumor*"[MeSH Terms]) OR "breast neoplasms"[MeSH Terms] OR "breast neoplasms"[MeSH Terms] OR "breast neoplasms"[MeSH Terms]) AND ("metastatic"[Text Word] OR "palliative"[Text Word] OR "advance*"[Text Word] OR "relapse*"[Text Word]) AND ("hormone receptor"[Text Word] OR "hormone receptor positive"[Text Word] OR "endocrine receptor positive"[Text Word] OR "endocrine receptor"[Text Word] OR "estrogen receptor positive"[Text Word] OR "estrogen receptor"[Text Word] OR "er positive"[Text Word] OR "progesterone receptor"[Text Word] OR "progesterone receptor positive"[Text Word] OR "pr positive"[Text Word]) AND ("drug therapy"[MeSH Terms] OR "immunotherapy"[MeSH Terms] OR ("tamoxifen"[MeSH Terms] OR "anastrozole"[MeSH Terms] OR "letrozole"[MeSH Terms] OR "exemestane"[Supplementary Concept] OR "toremifene"[MeSH Terms] OR "endocrine therapy"[Text Word] OR "hormone therapy"[Text Word] OR "selective estrogen receptor modulators"[MeSH Terms] OR "SERD"[Text Word] OR "SERM"[Text Word] OR "selective estrogen receptor downregulator"[Text Word] OR "fulvestrant"[MeSH Terms]) OR ("phosphoinositide 3 kinase inhibitors"[MeSH Terms] OR "alpelisib"[Supplementary Concept] OR "2 3 2 1 isopropyl 3 methyl 1h 1 2 4 triazol 5 yl 5 6 dihydrobenzo f imidazo 1 2 d 1 4 oxazepin 9 yl 1h pyrazol 1 yl 2 methylpropanamide"[Supplementary Concept]) OR ("mtor inhibitors"[MeSH Terms] OR "everolimus"[MeSH Terms]) OR ("angiogenesis inhibitors"[MeSH Terms] OR "bevacizumab"[MeSH Terms]) OR "antineoplastic agents"[MeSH Terms] OR ("cyclin dependent kinase inhibitor proteins"[MeSH Terms] OR "palbociclib"[Supplementary Concept] OR "abemaciclib"[Supplementary Concept] OR "ribociclib"[Supplementary Concept]))	4,910	09:58:04

	Concept])) OR (abemaciclib[Supplementary Concept])) OR (ribociclib[Supplementary Concept])))					
17	((((((drug therapy[MeSH Terms]) OR (immunotherapy[MeSH Terms])) OR ((((((((((citrate, tamoxifen[MeSH Terms]) OR (anastrozole[MeSH Terms])) OR (letrozole[MeSH Terms])) OR (exemestane[Supplementary Concept])) OR (toremifene[MeSH Terms])) OR (endocrine therapy[Text Word])) OR (hormone therapy[Text Word])) OR (selective estrogen receptor modulator[MeSH Terms])) OR (SERD[Text Word])) OR (SERM[Text Word])) OR (selective estrogen receptor downregulator[Text Word])) OR (fulvestrant[MeSH Terms]))) OR (((Phosphoinositide-3 Kinase Inhibitors[MeSH Terms]) OR (Alpelisib[Supplementary Concept])) OR (Taselisib[Supplementary Concept]))) OR ((MTOR Inhibitors[MeSH Terms]) OR (everolimus[MeSH Terms]))) OR ((Angiogenesis Inhibitors[MeSH Terms]) OR (Bevacizumab[MeSH Terms]))) OR (Antineoplastic Agents[MeSH Terms])) OR ((((Cyclin-Dependent Kinase Inhibitor Proteins[MeSH Terms]) OR (palbociclib[Supplementary Concept])) OR (abemaciclib[Supplementary Concept])) OR (ribociclib[Supplementary Concept]))	Publication Date		"drug therapy"[MeSH Terms] OR "immunotherapy"[MeSH Terms] OR "tamoxifen"[MeSH Terms] OR "anastrozole"[MeSH Terms] OR "letrozole"[MeSH Terms] OR "exemestane"[Supplementary Concept] OR "toremifene"[MeSH Terms] OR "endocrine therapy"[Text Word] OR "hormone therapy"[Text Word] OR "selective estrogen receptor modulators"[MeSH Terms] OR "SERD"[Text Word] OR "SERM"[Text Word] OR "selective estrogen receptor downregulator"[Text Word] OR "fulvestrant"[MeSH Terms] OR "phosphoinositide 3 kinase inhibitors"[MeSH Terms] OR "alpelisib"[Supplementary Concept] OR "2 3 2 1 isopropyl 3 methyl 1h 1 2 4 triazol 5 yl 5 6 dihydrobenzo f imidazo 1 2 d 1 4 oxazepin 9 yl 1h pyrazol 1 yl 2 methylpropanamide"[Supplementary Concept] OR "mtor inhibitors"[MeSH Terms] OR "everolimus"[MeSH Terms] OR "angiogenesis inhibitors"[MeSH Terms] OR "bevacizumab"[MeSH Terms] OR "antineoplastic agents"[MeSH Terms] OR "cyclin dependent kinase inhibitor proteins"[MeSH Terms] OR "palbociclib"[Supplementary Concept] OR "abemaciclib"[Supplementary Concept] OR "ribociclib"[Supplementary Concept]	21,66,524	09:57:49
16	(((((((breast cancer*[MeSH Terms]) OR (breast tumor*[MeSH Terms])) OR (breast neoplasm[MeSH Terms])) OR (breast carcinoma[MeSH Terms])) OR (breast tumour[MeSH Terms])) AND ((((metastatic[Text Word]) OR (palliative[Text Word])) OR (advance*[Text Word])) OR (relapse*[Text Word]))) AND (((((((((hormone receptor[Text Word]) OR (hormone receptor positive[Text Word])) OR (endocrine receptor positive[Text Word])) OR (endocrine receptor[Text Word])) OR (estrogen receptor positive[Text Word])) OR (estrogen receptor[Text Word])) OR (ER positive[Text Word])) OR (progesterone receptor[Text Word])) OR (progesterone	Publication Date		("breast cancer*"[MeSH Terms] OR (("breast"[MeSH Terms] OR "breast"[All Fields] OR "breasts"[All Fields] OR "breast s"[All Fields]) AND "tumor*"[MeSH Terms]) OR "breast neoplasms"[MeSH Terms] OR "breast neoplasms"[MeSH Terms] OR "breast neoplasms"[MeSH Terms]) AND ("metastatic"[Text Word] OR "palliative"[Text Word] OR "advance*"[Text Word] OR "relapse*"[Text Word]) AND ("hormone receptor"[Text Word] OR "hormone receptor positive"[Text Word] OR "endocrine receptor positive"[Text Word] OR "endocrine receptor"[Text Word] OR "estrogen receptor positive"[Text Word] OR "estrogen receptor"[Text Word] OR "er positive"[Text Word] OR "progesterone receptor"[Text Word] OR "progesterone receptor positive"[Text Word] OR "pr positive"[Text Word])	8,466	09:56:58

	receptor positive[Text Word])) OR (PR positive[Text Word])))					
15	(((Cyclin-Dependent Kinase Inhibitor Proteins[MeSH Terms]) OR (palbociclib[Supplementary Concept])) OR (abemaciclib[Supplementary Concept])) OR (ribociclib[Supplementary Concept])	Publication Date		"cyclin dependent kinase inhibitor proteins"[MeSH Terms] OR "palbociclib"[Supplementary Concept] OR "abemaciclib"[Supplementary Concept] OR "ribociclib"[Supplementary Concept]	28,157	09:56:20
14	Antineoplastic Agents[MeSH Terms]	Publication Date		"antineoplastic agents"[MeSH Terms]	4,74,792	09:54:48
13	(Angiogenesis Inhibitors[MeSH Terms]) OR (Bevacizumab[MeSH Terms])	Publication Date		"angiogenesis inhibitors"[MeSH Terms] OR "bevacizumab"[MeSH Terms]	34,178	09:53:55
12	(MTOR Inhibitors[MeSH Terms]) OR (everolimus[MeSH Terms])	Publication Date		"mtor inhibitors"[MeSH Terms] OR "everolimus"[MeSH Terms]	5,572	09:53:01
11	((Phosphoinositide-3 Kinase Inhibitors[MeSH Terms]) OR (Alpelisib[Supplementary Concept])) OR (Taselisib[Supplementary Concept])	Publication Date		"phosphoinositide 3 kinase inhibitors"[MeSH Terms] OR "alpelisib"[Supplementary Concept] OR "2 3 2 1 isopropyl 3 methyl 1h 1 2 4 triazol 5 yl 5 6 dihydrobenzo f imidazo 1 2 d 1 4 oxazepin 9 yl 1h pyrazol 1 yl 2 methylpropanamide"[Supplementary Concept]	9,568	09:52:11
10	((phosphotidyl 3 kinase inhibitor[MeSH Terms]) OR (alpelisib[Supplementary Concept])) OR (taselisib[Supplementary Concept])	Publication Date		"alpelisib"[Supplementary Concept] OR "2 3 2 1 isopropyl 3 methyl 1h 1 2 4 triazol 5 yl 5 6 dihydrobenzo f imidazo 1 2 d 1 4 oxazepin 9 yl 1h pyrazol 1 yl 2 methylpropanamide"[Supplementary Concept]	207	09:50:46
9	((Phosphoinositide-3 Kinase Inhibitors[MeSH Terms]) OR (alpelisib[MeSH Terms])) OR (taselisib[MeSH Terms])	Publication Date		"phosphoinositide 3 kinase inhibitors"[MeSH Terms]	9,434	09:49:39
8	(((((((((((citrate, tamoxifen[MeSH Terms]) OR (anastrozole[MeSH Terms])) OR (letrozole[MeSH Terms])) OR (exemestane[Supplementary Concept])) OR (toremifene[MeSH Terms])) OR (endocrine therapy[Text Word])) OR (hormone therapy[Text Word])) OR (selective estrogen receptor modulator[MeSH Terms])) OR (SERD[Text Word])) OR (SERM[Text Word])) OR (selective estrogen receptor downregulator[Text Word])) OR (fulvestrant[MeSH Terms])	Publication Date		"tamoxifen"[MeSH Terms] OR "anastrozole"[MeSH Terms] OR "letrozole"[MeSH Terms] OR "exemestane"[Supplementary Concept] OR "toremifene"[MeSH Terms] OR "endocrine therapy"[Text Word] OR "hormone therapy"[Text Word] OR "selective estrogen receptor modulators"[MeSH Terms] OR "SERD"[Text Word] OR "SERM"[Text Word] OR "selective estrogen receptor downregulator"[Text Word] OR "fulvestrant"[MeSH Terms]	51,810	09:47:59
7	((((((((((((endocrine therapy[Text Word]) OR (hormone therapy[Text Word])) OR (selective estrogen receptor modulator[MeSH Terms])) OR (tamoxifen[MeSH Terms])) OR (citrate, toremifene[MeSH Terms]))OR (anastrozole[MeSH Terms])) OR (letrozole[MeSH Terms])) OR (aromatase inhibitors[MeSH Terms])) OR (fulvestrant[MeSH Terms])) OR	Publication Date		"endocrine therapy"[Text Word] OR "hormone therapy"[Text Word] OR "selective estrogen receptor modulators"[MeSH Terms] OR "tamoxifen"[MeSH Terms] OR "toremifene"[MeSH Terms] OR "anastrozole"[MeSH Terms] OR "letrozole"[MeSH Terms] OR "aromatase inhibitors"[MeSH Terms] OR "fulvestrant"[MeSH Terms] OR "selective estrogen receptor downregulator"[Text Word] OR "SERD"[Text Word] OR	54,760	09:45:29

	(selective estrogen receptor downregulator[Text Word])) OR (SERD[Text Word])) OR (SERM[Text Word])) OR (exemestane[Supplementary Concept])			"SERM"[Text Word] OR "exemestane"[Supplementary Concept]		
6	(((((((((((endocrine therapy[Text Word]) OR (hormone therapy[Text Word])) OR (selective estrogen receptor modulator[MeSH Terms])) OR (tamoxifen[MeSH Terms])) OR (citrate, toremifene[MeSH Terms])) OR (exemestane[MeSH Terms])) OR (anastrozole[MeSH Terms])) OR (letrozole[MeSH Terms])) OR (aromatase inhibitors[MeSH Terms])) OR (fulvestrant[MeSH Terms])) OR (selective estrogen receptor donregulator[Text Word])) OR (SERD[Text Word])) OR (SERM[Text Word])	Publication Date		"endocrine therapy"[Text Word] OR "hormone therapy"[Text Word] OR "selective estrogen receptor modulators"[MeSH Terms] OR "tamoxifen"[MeSH Terms] OR "toremifene"[MeSH Terms] OR "anastrozole"[MeSH Terms] OR "letrozole"[MeSH Terms] OR "aromatase inhibitors"[MeSH Terms] OR "fulvestrant"[MeSH Terms] OR "SERD"[Text Word] OR "SERM"[Text Word]	54,599	09:42:52
5	immunotherapy[MeSH Terms]	Publication Date		"immunotherapy"[MeSH Terms]	3,19,626	09:33:24
4	drug therapy[MeSH Terms]	Publication Date		"drug therapy"[MeSH Terms]	14,75,057	09:33:06
3	(((((((((hormone receptor[Text Word]) OR (hormone receptor positive[Text Word])) OR (endocrine receptor positive[Text Word])) OR (endocrine receptor[Text Word])) OR (estrogen receptor positive[Text Word])) OR (estrogen receptor[Text Word])) OR (ER positive[Text Word])) OR (progesterone receptor[Text Word])) OR (progesterone receptor positive[Text Word])) OR (PR positive[Text Word])	Publication Date		"hormone receptor"[Text Word] OR "hormone receptor positive"[Text Word] OR "endocrine receptor positive"[Text Word] OR "endocrine receptor"[Text Word] OR "estrogen receptor positive"[Text Word] OR "estrogen receptor"[Text Word] OR "er positive"[Text Word] OR "progesterone receptor"[Text Word] OR "progesterone receptor positive"[Text Word] OR "pr positive"[Text Word]	96,285	09:31:58
2	(((metastatic[Text Word]) OR (palliative[Text Word])) OR (advance*[Text Word])) OR (relapse*[Text Word])	Publication Date		"metastatic"[Text Word] OR "palliative"[Text Word] OR "advance*"[Text Word] OR "relapse*"[Text Word]	14,22,626	09:30:04
1	((((breast cancer*[MeSH Terms]) OR (breast tumor*[MeSH Terms])) OR (breast neoplasm[MeSH Terms])) OR (breast carcinoma[MeSH Terms])) OR (breast tumour[MeSH Terms])	Publication Date		"breast cancer*"[MeSH Terms] OR (("breast"[MeSH Terms] OR "breast"[All Fields] OR "breasts"[All Fields] OR "breast s"[All Fields]) AND "tumor*"[MeSH Terms]) OR "breast neoplasms"[MeSH Terms] OR "breast neoplasms"[MeSH Terms] OR "breast neoplasms"[MeSH Terms]	3,59,552	09:29:23

S1.2. Embase

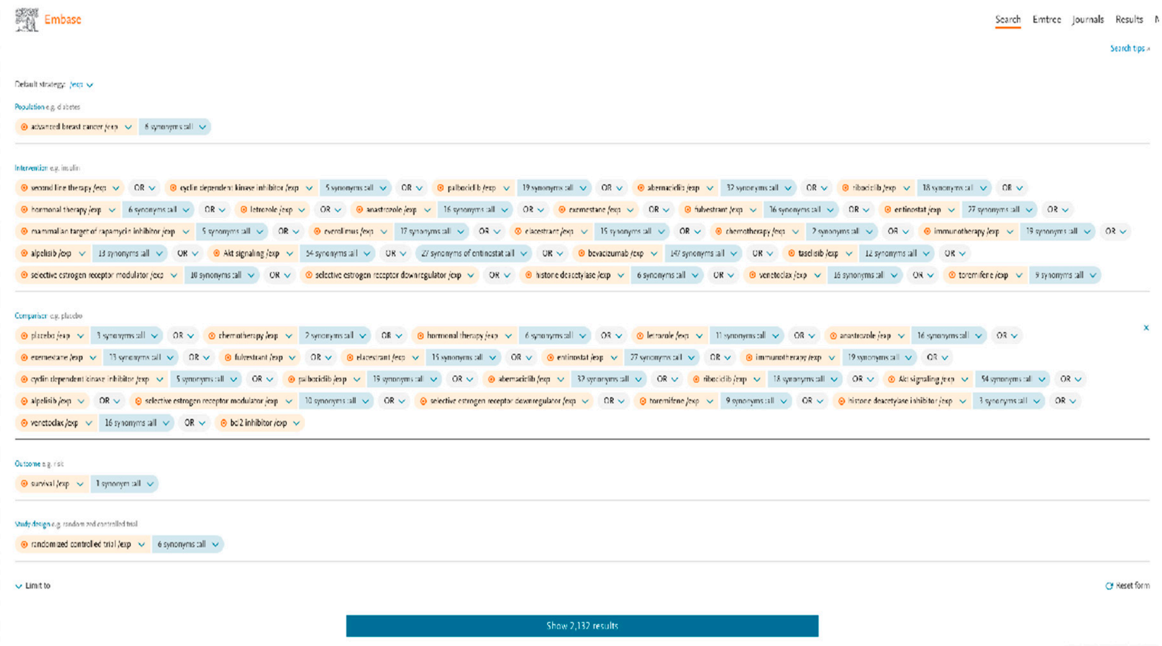

S1.3. Scopus

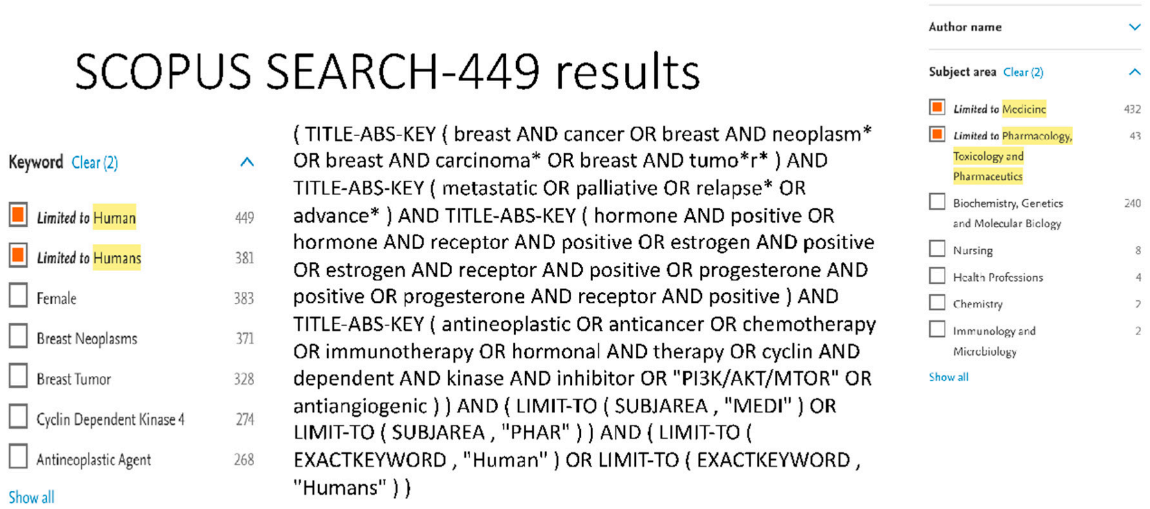

- With limits of human/humans and medicine and pharamacology studies

S1.4. Cochrane

#	Search	Field	Results
#1	MeSH descriptor: [Breast Neoplasms] explode all trees	MeSH	14801
#2	(hormone receptor positive):ti,ab,kw (Word variations have been searched)	Limits	4097
#3	(estrogen receptor positive):ti,ab,kw (Word variations have been searched)	Limits	2944
#4	progesterone receptor positive (Word variations have been searched)	Limits	1272
#5	estrogen receptor (Word variations have been searched)	Limits	5487
#6	progesterone receptor (Word variations have been searched)	Limits	2258
#7	ER positive (Word variations have been searched)	Limits	4470
#8	PR positive (Word variations have been searched)	Limits	3616
#9	hormone positive (Word variations have been searched)	Limits	10012
#10	metastatic	Limits	31755
#12	palliative (Word variations have been searched)	Limits	11009
#13	advance* (Word variations have been searched)	Limits	87622
#14	hormonal therapy (Word variations have been searched)	Limits	36542
#15	selective estrogen receptor modulators (Word variations have been searched)	Limits	1030
#16	selective estrogen receptor downregulators (Word variations have been searched)	Limits	42
#17	tamoxifen (Word variations have been searched)	Limits	5258
#18	anastrozole (Word variations have been searched)	Limits	1327
#19	letrozole (Word variations have been searched)	Limits	2477
#20	exemestane (Word variations have been searched)	Limits	1006
#21	toremifene (Word variations have been searched)	Limits	222
#22	fulvestrant (Word variations have been searched)	Limits	934

#	Search	Type	Results
#23	cyclin dependent kinase inhibitors (Word variations have been searched)	Limits	445
#24	cdk inhibitors (Word variations have been searched)	Limits	192
#25	palbociclib (Word variations have been searched)	Limits	526
#26	ribociclib (Word variations have been searched)	Limits	268
#27	abemaciclib (Word variations have been searched)	Limits	255
#28	chemotherapy (Word variations have been searched)	Limits	91517
#29	MeSH descriptor: [Drug Therapy] explode all trees	MeSH	148428
#30	MeSH descriptor: [Antineoplastic Agents] explode all trees	MeSH	13298
#31	MeSH descriptor: [Immunotherapy] explode all trees	MeSH	8777
#32	MeSH descriptor: [Selective Estrogen Receptor Modulators] explode all trees	MeSH	415
#33	MeSH descriptor: [Angiogenesis Inhibitors] explode all trees	MeSH	1380
#34	MeSH descriptor: [Bevacizumab] explode all trees	MeSH	2253
#35	MeSH descriptor: [MTOR Inhibitors] explode all trees	MeSH	1610
#36	MeSH descriptor: [Everolimus] explode all trees	MeSH	1637
#37	MeSH descriptor: [Phosphoinositide-3 Kinase Inhibitors] explode all trees	MeSH	29
#38	alpelisib (Word variations have been searched)	Limits	111
#39	taselisib (Word variations have been searched)	Limits	46
#40	MeSH descriptor: [Histone Deacetylase Inhibitors] explode all trees	MeSH	69
#41	entinostat (Word variations have been searched)	Limits	64
#42	buparlisib (Word variations have been searched)	Limits	67
#43	#10 OR #11 OR #12 OR #13 (Word variations have been searched)	Limits	150908
#44	#2 OR #3 OR #4 OR #5 OR #6 OR #7 OR #8 OR #9	Limits	14975
#45	#14 OR #15 OR #16 OR #17 OR #18 OR #19 OR #20 OR #21 OR #22 OR #23 OR #24 OR #25 OR #26 OR #27 OR #28 OR #29 OR #30 OR #31 OR #32 OR #33 OR #34 OR #35 OR #36 OR #37 OR #38 OR #39 OR #40 OR #41 OR #42	Limits	323086
#46	#1 AND {OR #10-#13} AND {OR #2-#9} AND {OR #14-#42}	Limits	1106

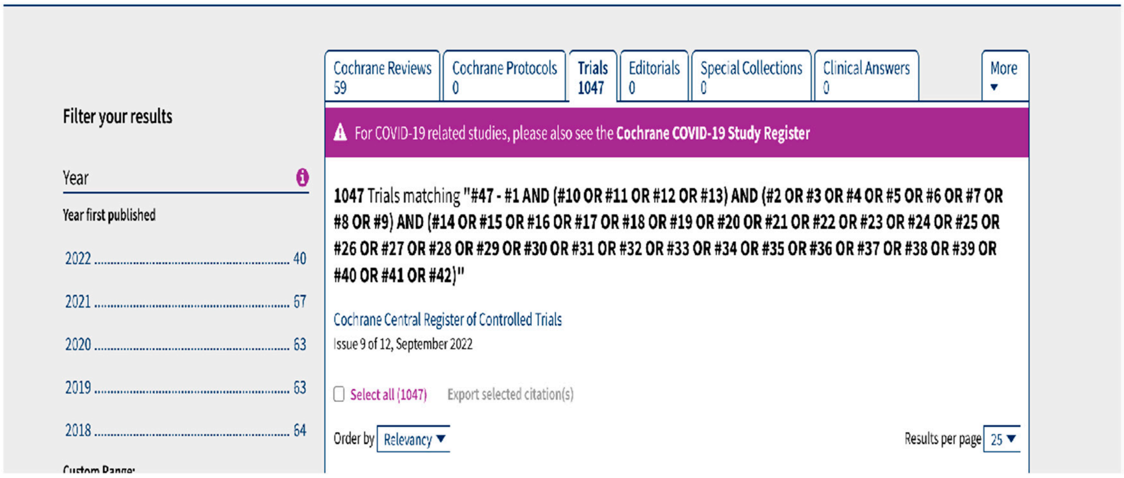

S1.5. Clinicaltrials.gov

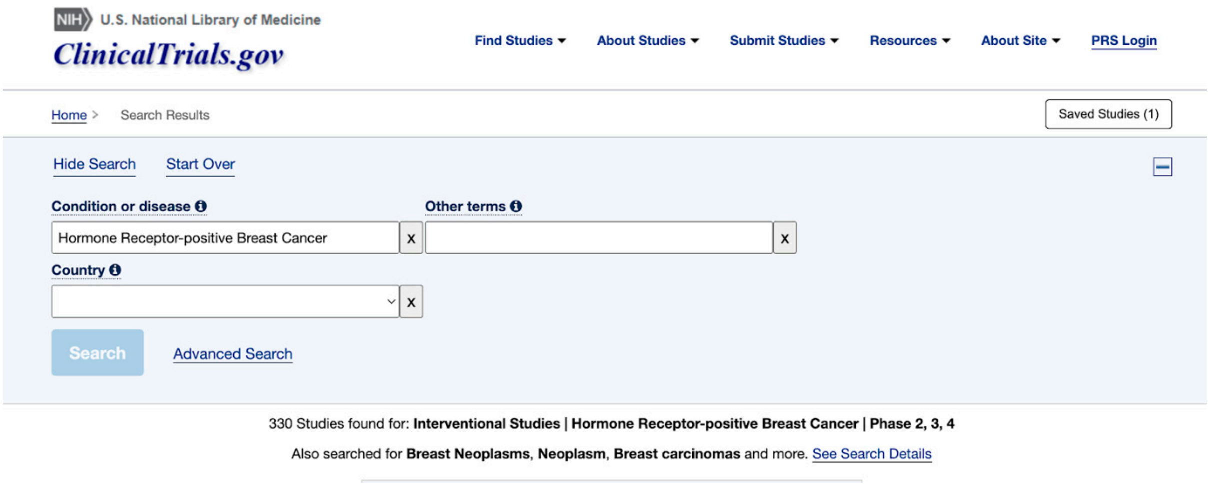

Supplemental tables

No	Study	Pt Population, N	Intervention	ORR/CBR	PFS	OS	AE	QoL/Comments
1.	NCT03401385 (TROPION-Pantumour01), phase II	HR+HER2- MBC post median 5 lines of therapy 95% had prior CDK 4/6i , n=41	Dato-DXD	29% /85%	-	-	15% had serious TEAEs	SABCS 2022 PD13-08, full paper awaited
2.	Coombes et al[58], phase II,2022	HR+HER2- MBC post Ai and prior CDK 4/6i , n=31	Samuraciclib (CDK7 inh)	-/ 35%	-	-	Grade ≥3 diarrhea (19%), nausea(10%), vomitive (3%), fatigue(3%)	Full paper awaited
3.	Hamilton et al[59], phase II	HR+HER2- MBC,85% CDK 4/6i exposed, 3 median lines of therapy, n=94	H3B-6545 (SERCA)	17% /32%	5.1m	-	Gr≥ 2 anemia (19%), nausea (17%), fatigue (16%)diarrhea (12%), creatinine clearance dec(39%)lymphopenia (37%), ALT/AST inc (14%)bilirubin inc (12%)	Full paper awaited
4.	Bardia et al 2021[60] (TRINITI) , phase I/II	HR+ HER2- MBC with PD after up to 3 lines of therapy incl CDK 4/6i, n=95	Ribociclib + exemestane + everolimus	8.4% /41%	5.7m	-	Gr≥3, Neutropenia(51%), Anemia (9.6%),hyperglycemia (6.7%), Hypophosphatemia (5.8%), stomatitis (2.9%)	-

5.	Seth et al[61], 2022 (TAKTIC), Phase IB	HR+/HER2- MBC; with PD on 1st line; up to 2 prior lines of chemotherapy, 84% post CDK 4/6i, n=77(A-19/B-16/C-42)	Arms:<br>A. Ipat+ AI<br>B. Ipat+ fulv<br>C. Ipat+ Fulv+ palbociclib	-/48%	5.5m	24.5m	Grade ≥3 neutropenia (50.6%), leukopenia (19.5%), diarrhea (14/3%), transaminitis (9.1%), lymphopenia (7.8%), rash (7.8%), and thrombocytopenia (3.9%)	Interim results
6.	AMEERA-1, phase I/II Part B[62]<br><br>Part C and D[63]	HR+/HER2- MBC, 1 CDK4/6i allowed, n=46<br><br>HR+/HER2- MBC, CDK4/6i allowed, n=39	Amcenestrant(SAR439859-SERD)<br><br>Amcenestrant + palbociclib	10.9%/28.3%<br><br>32.4%/73.5%	-	-	Grade ≥3 ALT/AST inc(6.1%), fatigue(4%) Nausea, vomiting and diarrhea-2% each<br><br>56.4% gr≥3 neutropenia, no non hemat gr≥3 toxicities	Interim results, full paper awaited
7.	Kalinsky et al[64],2022, phase I	HR+/HER2- MBC, 1 CDK4/6iallowed in 68%	ZN-c5 (SERD)	-/31%	3.8m	-	Gr≥3: 2/56 GGT inc, hyponatremia (2/56); no Grade 4 TEAEs	Full paper awaited
8.	Schott et al[65], 2023, VERITAC study, phase I/II	HR+/HER2- MBC, PD post CDk4/6i	ARV-471, PROTAC (Proteolysis Targeting Chimera)<br><br>200mg (n=35)<br><br>500mg (n=36)	 <br>-/37.1%<br><br>-/38.9%	-	-	Gr≥3 TRAE in 7% cases overall	Full paper awaited
9.	Patel et al[66], 2022, Phase I/II	HR+/HER2- MBC, 1 CDK4/6iexposed, n=40	OP-1250, (CERAN/SERD)	In the anticipated RP2D range, ORR-18% &	-	-	Gr ≥3 in 24.3% pts, Nausea and vomiting (2.7% each), neutropenia (8.1%)	Full paper awaited

				CBR-38%				
10.	Wesolowski et al[67], 2023,phase Ib/II	HR+ HER2- MBC exposed to CDK 4/6i post ≥2 lines of treatment (subset C & D), n= C32/ D 27	Gedatolisib (PI3K &mTOR inh) + palbociclib + fulvestrant	ORR C/D:36% /63% No CBR	C/D: 5.1m/ 12.9m	-	Gr≥3 in all subsets: neutropenia (63%), stomatitis (27%), rash (20%), fatigue (11%) and hyperglycemia (7%)	Full paper awaited
11.	Pistilli et al[68], 2020, phase II	HR+, HER2 low (IHC 1+/IHC 2+ with negative FISH) MBC post CDK 4/6i and <4 lines ET & <3 lines CT, n=48	Zenocutuzumab +ET (bispecific Ab targeting HER2 and HER3)	-/45%	-	-	Gr ≥3 Asthenia/fatigue(2%)	Full paper awaited
12.	Bardia et al 2021[69], phase Ib	HR+, HER2- MBC post AI & CDK 4/6i (group 2, n=17)	Ribociclib+ everolimus + exemestane	-/14.3%	1.9m	-	Gr≥3 total (76.5%), Anemia, neutropenia (35.3%), Leucopenia (29.4%)	-
13.	Rugo et al[70,71] BYLieve, phase II	PIK3CA-mutant, HR+, HER2– MBC PD on/after prior CDK4/6i combination therapy. Cohort A, n=127 Cohort B, n=126	Alpelisib + fulv (cohort A) Alpelisib + AI (cohort B)	A: 21% /55% B: 15.7% / 32.2%	A:7.3m B:5.7m	A: 17.3m	Cohort A: Gr≥ 3 AE hyperglycemia (28%), rash (9%), rash maculopapular (9%) & diarrhea (6%) Cohort B: hyperglycemia (25.4%), rash (9.5%), rash maculopapular (7.9%)	Non-comparative phase II study with 2 arms
14.	Albanell et	HR+, HER2– MBC PD	Palbociclib + different ET	-/34.4%	2.6m	-	Gr≥3: All 51.6%,	-

	al[72],2023. BioPER, phase II	on/after prior Palbociclib + ET, n=33. Two lines of prior ET and 1 line of CT allowed.					Neutropenia (42.4%),leucopenia (6.1%) anemia (3%)	
15.	Jhaveri et al[73], 2021, phase I/Ib	HR+, HER2– MBC, PD after ET. CDK 4/6i allowed in 49.2%, n=199 (A, B: 78 each C: 43) PI3K/AKT inh not allowed in arm C	A: LSZ102 (SERD) B: LSZ102 + ribociclib C: LSZ102 + Alpelisib	A: 1.3% /7.8% B: 16.9% /35.1 % C: 7% /20.9 %	A: 1.8m B: 6.2m C: 3.5m	-	Gi toxicity most common for all grades. For Gr≥3, were related to ribociclib in arm B(leukopenia, neutropenia, AST inc) and alpelisib in arm C(hyperglycemia, rash and loss of appetite)	PFS similar whetHER exposed to CDK4/6i or not
16.	Lim et al[74], 2022, phase I/II	HR+, HER2– MBC, PD after ET. CDK 4/6i allowed in n=25/31 pts.	Lenvatinib + letrozole	20%/ 52% (exposed to CDk4/6i)	-	-	-	-
17.	Mayer et al[75] 2021, phase Ib	HR+, HER2– MBC, PD after CDK 4/6i with FGFR1-4 amplification, n=26	erdafitinib(pan FGFR TKI) + palbociclib + fulvestrant	-/28%	3m	-	Gr ≥3: FN(5%), colitis(3.8%), thromboembolism(3.8%), elevation of transaminases(3.8%)	Better response with FGFR1 & FGFR3 amplification
18.	Pimentel et al[76] 2022, phase II. LUZERN study	HR+, HER2– MBC with gBRCAm (cohort A), gBRCA wild type /HRD(cohort B),	Niraparib + AI	-	5.3m	-	50% had Gr≥3 neutropenia	Interim analysis

		83% post CDK 4/6i, n=6						
19.	Lim et al[77], 2020, Phase Ib	HR+, HER2– MBC, cohort A post CDK 4/6i , n =39(cohort A)	GDC-9545 (SERD) ± LHRH agonist	-/55%	-	-	Cohort A: gr≥3 1 case each of fatigue, transaminase inc & diarrhea. Cohort B	-
20.	Schwartzberg et al[78] 2021, CONTESSA-2[79], phase II	HR+, HER2- MBC, prior taxane not allowed, n=150, 52% are post CDK 4/6i	Tesetaxel (oral taxane) + reduced dose capecitabine	51%/-	12.9m	-	Gr ≥3: neutropenia (74.0%), leukopenia (10.7%), hypokalemia (7.3%), anemia (6.7%), hand-foot syndrome (6.0%) and diarrhea (5.3%), FN(4.7%), neuropathy(2%)	Data for CDK 4/6i exposed not available separately, full paper awaited
21.	Prat et al[80], 2023, TATEN trial, phase II	HR+/HER2-negative, PAM50 non-luminal MBC PD post CDk4/6i,no CT in metastatic setting n=15	Pembrolizumab and paclitaxel	53.3% / 86.6%	7.5m	-	Gr ≥3 AE in 53.3%	Interim results
22.	SUMMIT [81] , phase II basket trial	HR+ MBC with HER 2 mutations, n=46(breast cancer cohort), 67% got prior CDK 4/6i	Neratinib + trastuzumab + fulvestrant	40% / 47%	8.3m	-	Gr 3 diarrhea (33%),no Gr 4 diarrhea	Ongoing study
23.	Damodaran et	HR+MBC PD post 1-	lasofoxifene +	33.3% /	13.9m	-	MC AE: diarrhea,	Ongoing study

	al[82], 2022 ELAINE 2, Phase II	2 lines of ET, with a ESR1 mutation, n=29 (28 pts got CDK 4/6i in prior line)	abemaciclib	62.1%			nausea, and leukopenia 2 d/c tt due to AE	
24	Tao et al[83], 2023, phase II	HR+/HER2-negative with PD on 1st line palbociclib + AI, n=58	Palbociclib + fulvestrant	-	3.7m	-	-	Ongoing study
25.	Bedard et al[84], 2023, phase Ib/II(Simon 2 stage design)	HER2- MBC post >1 line of non taxane CT. 92% HR+ HER2- MBC 81% post CDK 4/6i	CFI-402257 (inhibitor of TTK protein kinase) +paclitaxel	8% / 54.6%		-	Gr≥3 neutropenia (83.5%), lymphopenia (41%) and anemia (14%), FN(8.1%), skin infection (2.7%)	Trial closed, did not meet prespecified threshold for stage 2
26	Miller et al[85] 2023, phase II	HR+ MBC post 2-4 lines of tt including a CDK 4/6i(arm A),n=60	praluzatamab ravtansine (CX-2009)	14.9% / 40.4%	11.4weeks	-	Gr≥3 ocular (15%), neuropathic(10%)	Full paper awaited
27	Haddad et al[86], TBCRC 041 trial, phase II with 2 arms (that are not compared to one another)	HR+ HER 2- MBC, PD on CDK 4/6i, n=	Alisertib + fulvestrant<br><br>Alisertib	ORR:20%<br>and<br>19.6%<br>CBR: 28.95 v 41.3%	5.4m v 5.6m	19.8m v 22.7m	Neutropenia (42.2% v 43.4%), leukopenia (31.1% v 17.4%), and anemia (8.9% v 19.6%), lymphopenia (15.6% v 4%), fatigue (11.1% v 0%)	Although RCT, results reported as independent cohorts

Abbreviations:
AE, adverse events; AI, aromatase inhibitor; AKT, protein kinase B; ALT, alanine transaminase; AST, aspartate aminotransferase; CBR, clinical benefit rate; CDK 4/6i, cyclin dependent kinase 4/6 inhibitor; CERAN, Complete Estrogen Receptor Antagonist; CT, chemotherapy; Dato-DXD, Datopotamab deruxtecan; ET endocrine therapy; FGFR, fibroblast growth factor receptor; FN, febrile neutropenia; Fulv, fulvestrant; gBRCAm, germline breast cancer gene mutated; GGT, gamma-glutamyl transferase; Gr, grade; HER2-, human epidermal growth factor receptor 2 negative;HER3, human epidermal growth factor receptor 3; HR+, hormone receptor positive; HRD, homologous recombination deficiency; inc, increase; inh, inhibitor; Ipat, ipatasertib; LHRH, luteinizing hormone-releasing hormone; MBC, metastatic breast cancer; MC, most common; mTOR, Mammalian target of rapamycin; OR, odds ratio; ORR overall response rate; OS, overall survival; PD, progressive

disease; PI3KCA, phosphatidylinositol-4,5-bisphosphate 3-kinase catalytic subunit alpha; PFS, progression free survival, QoL, quality of life; SABCS, San Antonio breast cancer symposium; SERCA, Selective estrogen receptor covalent antagonist; SERD, selective estrogen receptor degraders; TEAE, treatment emergent adverse events; TKI, tyrosine kinase inhibitor; TRAE, treatment related adverse events; TTK threonine and tyrosine kinase

Table S1: Summary of prospective single-arm studies

Study	Phase and design	Intervention	control	Results	Comments
Sonnenblick et al, 2021 MORPHEUS [87]	Ib/II, 2L/3L HR+ MBC, exposed to CDK 4/6i, CT naive	Atezolizumab + Entinostat (n=15)	Fulvestrant (n=14)	ORR 6.7% v 0%<br>PFS 1.8m v 1.8m<br>CBR 40.0% v 21.4%	PDL1 did not correlate with response.

Goetz et al[88], 2022 ELAINE 1	II, HR+/HER2-/ESR1 mutated MBC PD on AI plus CDK4/6i	Lasofoxifene (SERM) (n=52)	Fulvestrant<br><br>(n=51)	PFS 6.04m v 4.04m;<br>HR 0.7;<br>95% CI 0.45 to 1.13<br>ORR<br>13.2% vs 2.9%, p 0.12<br>CBR<br>36.5% v 21.6%,	All results statistically NS
Goetz et al [89], 2020	II, ER+ HER 2- MBC , PD post ET,CDK 4/6i allowed, tamoxifen not allowed	Endoxifen (n=40, 42.5% post CDK 4/6i)	Tamoxifen (n=37, 29.7% post CDK 4/6i	PFS 130days v 42 days;<br>HR 0.8; 95% CI<br>0.49 to 1.22, p 0.309	-
Jimenez et al[90] 2022, acelERA trial	II, HR+ HER2- MBC for 2L/3L tt, CDK 4/6i allowed	Giredestrant (SERD) (n=151, 43% post CDK 4/6i)	ET (n=152,41% post CDK 4/6i)	PFS<br>5.6m v 5.4m;<br>HR 0.8; 95% CI 0.6 to 1.1, p 0.18<br>ORR 13% v 7%	-
Mayer et al[91], 2022, PACE trial	II, HR+/HER2- MBC, PD on AI and any CDK4/6i, only 1 line of CT allowed for MBC	Three arms with 1:2:1 randomisation:<br><br>Fulvestrant + Palbociclib (FP) (n=111)<br><br>Fulvestrant + Palbociclib + Avelumab (FPA) (n=54)	Fulvestrant(F) (n=55)	PFS of FP v F: 4.6m v 4.8 m; HR 1.11; 90% CI 0.79 to1.5, p 0.62<br><br>PFS of FPA v F:8.1m v 4.8m;<br>HR 0.75; 90% CI 0.50 to 1.12, p 0.23<br><br>ORR<br>13% (FPA)<br>v 9%(FP)<br>v 7.3%(F)	PFS of FPA v F , ORR and AE
SERENA-2[92]	II, HR+HER2- MBC, CDK4/6i allowed	A: Camizestrant (SERD) 75mg (n=74)<br><br>B: Camizestrant 150mg (n=73)	C: Fulvestrant<br><br>(n=173)	PFS<br>A<br>7.2m; HR 0.58; 95% CI 0.41to 0.81, p 0.01<br><br>B<br>7.7m; HR 0.67; 95% CI	ORR<br>15.7%<br><br>v<br><br>20%<br><br>v<br><br>11.8%

				0.48 to 0.92,<br>p 0.02<br><br>C<br>3.7m<br><br>*CDK 4/6i exposed:*<br><br>A<br>5.5m; HR; 0.49; 95% CI 0.31 to 0.75<br>B<br>3.8m; HR; 0.68; 95% CI 0.44 to 1.04<br><br>C<br>2.1m	CBR<br><br>47.3%<br><br>v<br><br>49.3%<br><br>39.1%
PALMIRA[93]	II, HR+ HER2- MBC post palbociclib	Palbociclib + ET (based on prior ET)<br>(n=136)	ET<br>(n=62)	PFS 4.2m v 3.6m<br>HR 0.8; 95% CI 0.6–1.1,<br>p 0.21<br><br>ORR<br>6.4% v<br>2.3% CBR<br>33.0% v<br>29.5%	-
2L/3L, second line or third line therapy; AI, aromatase inhibitor; CBR, clinical benefit rate; CDK 4/6i, cyclin dependent kinase 4/6 inhibitor; CT, chemotherapy; ET endocrine therapy; HER2-, human epidermal growth factor receptor 2 negative; HR, hazard ratio; HR+, hormone receptor positive; MBC, metastatic breast cancer; mTOR, ORR overall response rate; OS, overall survival; PD, progressive disease; PFS, progression free survival PI3KCA, phosphatidylinositol-4,5-bisphosphate 3-kinase catalytic subunit alpha; PFS, progression free survival; SABCS, San Antonio breast cancer symposium; SERD, selective estrogen receptor degraders; tt treatment.					

Table S2: RCTs with some results that include CDK 4/6i exposed pts; their full-text publication is awaited

Trial	Design/patient population	Intervention	Control	Comments
NCT04494425/<br>DESTINY-breast 06	Phase III, RCT, HR+ HER2 low MBC post CDK 4/6i, with	T-DXd	TPC	-

	disease PD within 6m of first line CDK 4/6i+ ET in metastatic setting/ PD≥2 lines of ET			
NCT03939897	Phase I/II, HR+ HER2- MBC, endocrine resistant, PI3K inhibitor naïve.	Copanlisib + fulvestrant + abemaciclib	fulvestrant + abemaciclib	CDK 4/6i allowed in phase I part of the study and as prior adjuvant tHERapy in phase II
NCT04895358/KEYNOTE B49	Phase III RCT, HR+ HER2- MBC Group 1 and 2b are post CDK 4/6i. PI3K and mTOR inhibitors allowed	Pembrolizumab + CT	Placebo + CT	Needs to be a CT candidate. PD-L1 CPS ≥1
NCT05104866 (TROPION-Breast01)	Phase III RCT of Datopotamab deruxtecan vs TPC in HR+HER2- MBC post 1-2 lines of CT	Datopotamab deruxtecan	TPC	previous lines of chemotHERapy (1 vs. 2), prior use of CDK4/6 inhibitors (Yes vs. no) & geographic region of participant.
NCT05306340 (evERA Breast Cancer)	Phase III RCT, HR+ HER2- MBC post CDK4/6i	Giredestrant + everolimus	TPET+ everolimus	Prior oral SERD, CERAN, PROTAC not allowed. Fulvestrant allowed with last dose ≥1m
NCT04802759	Phase Ib/II, randomised umbrella study HR+ HER2- MBC post CDK4/6i (cohort 1)	Giredestrant + abemaciclib<br>Giredestrant + ipatasertib<br>Giredestrant + inavolisib<br>Giredestrant + ribociclib	Giredestrant	Multiple treatment arms, primary endpoint is ORR

		Giredestrant + everolimus		
		Giredestrant + samuraciclib		
		Giredestrant + atezolizumab		
		Giredestrant + abemaciclib + atezolizumab		
NCT04215146/ Bracelet-1	Phase II RCT, three cohort study.<br>HR+ HER2- MBC post CDK4/6i	Pelareorep + paclitaxel + avelumab<br>Pelareorep + paclitaxel	paclitaxel	Oncolytic virus with a potential late onset adaptive immune response
NCT05169567/postMONARCH	Phase III RCT, HR+ HER2- MBC post CDK4/6i + AI	Abemaciclib + fulvestrant	Placebo + fulvestrant	Prior fulvestrant not allowed
NCT05079360	Phase II RCT, HR+ HER2- MBC post CDK4/6i, AI and fulvestrant	Sabizabulin	Exemestane/exemestane + everolimus/ SERM	Novel microtubule disrupter, also has anti-viral activity.
NCT05038735/EPIK B5	Phase III RCT, HR+ HER2- MBC post CDK4/6i, with mutated PI3KCA	Alpelisib + fulvestrant	Placebo + fulvestrant	-
NCT05305924	Phase II RCT, HR+ HER2- MBC post CDK4/6i	Fulvestrant alone for 1m□ abemaciclib +fulvestrant	Abemaciclib + fulvestrant	Tests whetHER fulvestrant run-in restores drug sensitivity
NCT04650581/FINER	Phase III RCT, HR+ HER2- MBC post CDK4/6i	Ipatasertib + fulvestrant	Placebo + fulvestrant	No prior fulvestrant or PI3K/AKT/mTOR inhibitors
NCT05159778.	Phase 2, Simon's 2-Stage study of MBC pts who have progressed through prior ET with >1 CDK4/6 inhibitor	Odetiglucan + Pembrolizumab	-	Odetiglucan is a novel beta glucan, acts as a pathogen-associated molecular pattern (PAMP)

				immune modulator
NCT04305834	Phase IIa study in HR+HER2-MBC older than 70 years of age and post ET +/- ribociclib/palbociclib	abemaciclib	-	Primary outcome is AE
NCT04699630	Phase 2 study of U3-1402 (Patritumab Deruxtecan), in which parts A and B enroll HR+HER2-MBC post CDK4/6i	U3-1402	-	U3-1402 is a ADC against HER3, primary outcome ORR
NCT04965766 (ICARUS-Breast)	Phase 2 study of U3-1402, in HR+HER2-MBC resistant to CDK4/6i	Patritumab Deruxtecan (U3-1402)	-	primary outcome ORR
NCT04305834	Phase II, ≥70 years, HR+ HER 2-MBC post palbociclib or ribociclib	abemaciclib	-	Primary outcome is grade 3+ toxicity
NCT05536128	Phase II, Umbrella study , in HR+ HER2-MBC post CDK 4/6i	Fulvestrant + Olaparib	-	-
NCT04762979	Phase II, HR+ HER2- MBC post CDK 4/6i, PI3KCA mutant	Alpelisib + fulvestrant/AI	-	-
NCT03326674 (CONTESSA)	Phase III RCT in HR+HER2- MBC treated with taxane in (neo)adjuvant setting	Tesetaxel + Capecitabine	Capecitabine	Tesetaxel is a novel oral taxane
NCT04738292 (SMILE)	Phase II study with HR+HER2-MBC progressed on ET +/- CDK4/6i, mTOR	Onapristone + fulvestrant	-	Onapristone is a antiprogestin

	inhibitors and 1 line of CT			
NCT05191914	Single arm study in HR+HER2- MBC resistant to CDK 4/6i +AI	Chidamide + fulvestrant	-	Chidamide is a histone deacetylase inhibitor
NCT04024436 (FOENIX-MBC2)	Phase 2 study with HR+HER2- MBC with FGFR1 amplification, post 1-2 ET +/- CDK 4/6i, ≤1 CT CDK4/6 inhibitor (if eligible)	Futibatinib + fulvestrant	-	FGFR1 amplification occur in 10% of MBC
NCT04460430 (SOLTI-1718 NEREA)	Phase 2 single arm Simon 2 stage design, locally advanced/MBC HR+ HER2-, with HER2-enriched(HER2-E)	Neratinib +ET	-	HER2-E subtype represents approximately 6.6-11.0% of HR+/HER2-BC
NCT04059484	Phase II RCT, HR+HER2- MBC progressed on ET +/- CDK4/6i	SAR439859	TPET	SAR439859 is an oral SERD
NCT04720664 (OASIS)	Phase II single arm in HR+HER2-MBC with ≤4 lines of tHERapy	SM-88 +MPS (methoxsalen, phenytoin, and sirolimus)	-	SM-88 is a dysfunctional tyrosine derivative. Primary outcome: ORR
NCT05286437 (LaPemERLA)	Phase II single arm, HR+HER2- MBC with at least 1 line of prior tHERapy	Pembrolizumab+ lenvatinib + Letrozole	-	-
NCT05181033	Phase II RCT of HR+HER2- MBC progressed on ET +/- CDK4/6i	Lenvatinib + letrozole	fulvestrant	-
NCT05501886 (VIKTORIA-1)	Phase III RCT with parallel assignment to 6 arms. HR+HER2- MBC progressed	Arm A: Gedatolisib + Fulvestrant +palbociclib<br>Arm B:<br>Gedatolisib + Fulvestrant<br>Arm C:		First three arms are PI3KCA WT and remaining

	on ET +/- CDK4/6i	fulvestrant<br>Arm D (mPI3KCA): Gedatolisib + Fulvestrant +palbociclib<br>Arm E(mPI3KCA): Fulvestrant +alpelisib<br>Arm F(mPI3KCA): Gedatolisib + Fulvestrant		are mPI3KCA. Primary outcome is PFS in Patients with PIK3CA WT and PIK3CA MT Breast Cancer
NCT04975308 (EMBER-3)	Phase III RCT HR+HER2- MBC progressed on ET +/- CDK4/6i	Imlunestrant + Abemaciclib	TPET	No CT/fulvestrant or otHER SERD allowed
NCT05392608 (SEQUEL-Breast)	Phase II single arm study, HR+HER2-MBC with mPI3KCA, must have taken CDK 4/6i in prior tHERapy	Alpelisib +fulvestrant	-	Last line of tHERapy must be fulvestrant
NCT04090398	HER2- MBC with bone metastases or non-bone only if:<br>≤5 visceral metastasis (≤4 cm in size) and asymptomatic Enlarged lymph nodes ≤4 cm	Radium Ra 223 Dichloride +paclitaxel	Paclitaxel	-
NCT05065411 (ENABLAR-2)	Phase III RCT in AR+ HR+ HER2- MBC with AR nuclear staining of ≥40%, PD post palbociclib +ET	Enobosarm + abemaciclib	Fulvestrant<br>Or AI depending on prior line	Enobosarm is a oral selective AR targeting agonis
ARTEST NCT04869943	Phase III RCT in AR+ HR+ HER2- MBC with AR nuclear staining of ≥40%, PD post CDK4/6i, fulvestrant and AI	Enobosarm	Physician's choice of exemestane ± everolimus or SERM)	-

Abbreviations: AI, aromatase inhibitor; AR, androgen receptor; CBR, clinical benefit rate; CDK4/6i, cyclin dependent kinase 4/6 inhibitor; CPS, combined positive score; CT, chemotherapy; ET, endocrine therapy; FGFR, Fibroblast growth factor receptor; HR, hazard ratio; HR+, hormone receptor positive; HER2-,human epidermal growth factor receptor2 negative; m, mutated; MBC, metastatic breast cancer; ORR, overall response rate; PI3KCA phosphatidylinositol-4,5-bisphosphate 3-kinase catalytic subunit alpha, PD, progressive disease; PFS, progression free survival; RCT, randomised controlled trial; SERD,

Selective estrogen receptor degrader; SERM, Selective estrogen receptor modulators; T-DXd, Trastuzumab deruxtecan; TPC, the physician's choice of chemotherapy; TPET, the physicians choice of ET

Table S3: Relevant ongoing studies in the post-CDK 4/6i domain

Supplemental figures legends

Figure S1a. Traffic plot for risk of bias assessment.<br>Red circle- high risk of bias<br>Yellow circle- some concerns<br>green circle- low risk of bias<br>blue question mark- no information
Figure S1b. Summary plot for risk of bias assessment<br>Red bar- high risk of bias<br>Yellow bar- some concerns

green bar- low risk of bias<br>blue bar- no information
Figure S2 : Forest plot showing the network meta analysis of progression free survival through network meta-analysis of oral Selective Estrogen Receptor Degraders to continuing ribociclib in mutated ESR1 patients
Figure S3: Forest Plot showing network meta-analysis of grade 3 and above adverse events for Selective Estrogen Receptor Degraders
Figure S4: Forest Plot showing network meta-analysis of progression free survival of all interventions compared to that of continuing ribociclib
Figure S5a: Forest plot of network meta-analysis comparison of Grade≥3 hematological toxicities vs ribociclib
Figure S5b: Forest plot of comparison network meta-analysis of Non-hematological grade≥3 adverse effects of all agents vs ribociclib.

58. Coombes C, Howell SJ, Krebs MG, Lord S, Kenny LM, Bahl A, et al. Abstract GS3-10: Study of s samuraciclib (CT7001), a first-in-class, oral, selective inhibitor of CDK7, in combination with fulvestrant in patients with advanced hormone receptor positive HER2 negative breast cancer (HR+BC). Cancer Research [Internet]. 2022 Feb 15 [cited 2022 Nov 18];82(4_Supplement):GS3-10. Available from: https://doi.org/10.1158/1538-7445.SABCS21-GS3-10

59. Hamilton EP, Wang JS, Pluard TJ, Johnston SRD, Morikawa A, Dees EC, et al. Phase I/II study of H3B-6545, a novel selective estrogen receptor covalent antagonist (SERCA), in estrogen receptor positive (ER+), human epidermal growth factor receptor 2 negative (HER2-) advanced breast cancer. JCO [Internet]. 2021 May 20 [cited 2022 Nov 20];39(15_suppl):1018–1018. Available from: https://ascopubs.org/doi/abs/10.1200/JCO.2021.39.15_suppl.1018

60. Bardia A, Hurvitz SA, DeMichele A, Clark AS, Zelnak A, Yardley DA, et al. Phase I/II Trial of Exemestane, Ribociclib, and Everolimus in Women with HR+/HER2- Advanced Breast Cancer after Progression on CDK4/6 Inhibitors (TRINITI-1). Clin Cancer Res. 2021 Aug 1;27(15):4177–85.

61. Wander SA, Keenan JC, Niemierko A, Juric D, Spring LM, Supko J, et al. Abstract PD13-07: PD13-07 Combination therapy with the AKT inhibitor, ipatasertib, endocrine therapy, and a CDK4/6 inhibitor for hormone receptor positive (HR+)/HER2 negative metastatic breast cancer (MBC): results from the phase I TAKTIC trial. Cancer Research [Internet]. 2023 Mar 1 [cited 2023 Mar 21];83(5_Supplement):PD13-07. Available from: https://doi.org/10.1158/1538-7445.SABCS22-PD13-07

62. Bardia A, Chandarlapaty S, Linden HM, Ulaner GA, Gosselin A, Cartot-Cotton S, et al. AMEERA-1 phase 1/2 study of amcenestrant, SAR439859, in postmenopausal women with ER-positive/HER2-negative advanced breast cancer. Nat Commun [Internet]. 2022 Jul 15 [cited 2023 Mar 22];13(1):4116. Available from: https://www.nature.com/articles/s41467-022-31668-8

63. Chandarlapaty S, Linden HM, Neven P, Petrakova K, Bardia A, Kabos P, et al. Abstract P1-17-11: Updated data from AMEERA-1: Phase 1/2 study of amcenestrant (SAR439859), an oral selective estrogen receptor (ER) degrader (SERD), combined with palbociclib in postmenopausal women with ER+/HER2- advanced breast cancer. Cancer Research [Internet]. 2022 Feb 15 [cited 2022 Nov 20];82(4_Supplement):P1-17–11. Available from: https://doi.org/10.1158/1538-7445.SABCS21-P1-17-11

64. Kalinksy K, Abramson V, Chalasani P, Linden HM, Alidzanovic J, Layman RM, et al. Abstract P1-17-02: ZN-c5, an oral selective estrogen receptor degrader (SERD), in women with advanced estrogen receptor-positive (ER+)/human epidermal growth factor receptor 2 negative (HER2-) breast cancer. Cancer Research [Internet]. 2022 Feb 15 [cited 2022 Nov 20];82(4_Supplement):P1-17–02. Available from: https://doi.org/10.1158/1538-7445.SABCS21-P1-17-02

65. Schott AF, Hurvitz S, Ma C, Hamilton E, Nanda R, Zahrah G, et al. Abstract GS3-03: GS3-03 ARV-471, a PROTAC® estrogen receptor (ER) degrader in advanced ER-positive/human epidermal growth factor receptor 2 (HER2)-negative breast cancer: phase 2 expansion (VERITAC) of a phase 1/2 study. Cancer Research [Internet]. 2023 Mar 1 [cited 2023 Mar 29];83(5_Supplement):GS3-03. Available from: https://doi.org/10.1158/1538-7445.SABCS22-GS3-03

66. Patel M, Alemany C, Mitri Z, Makower D, Borges V, Sparano J, et al. Abstract P1-17-12: Preliminary data from a phase I/II, multicenter, dose escalation study of OP-1250, an oral CERAN/SERD, in subjects with advanced and/or metastatic estrogen receptor (ER)-positive, HER2-negative breast cancer. Cancer Research [Internet]. 2022 Feb 15 [cited 2022 Nov 20];82(4_Supplement):P1-17–12. Available from: https://doi.org/10.1158/1538-7445.SABCS21-P1-17-12

67. Wesolowski R, Rugo H, Stringer-Reasor E, Han HS, Specht JM, Dees EC, et al. Abstract PD13-05: PD13-05 Updated results of a Phase 1b study of gedatolisib plus palbociclib and endocrine therapy in women with hormone receptor positive advanced breast cancer: Subgroup analysis by PIK3CA mutation status. Cancer Research [Internet]. 2023 Mar 1 [cited 2023 Mar 22];83(5_Supplement):PD13-05. Available from: https://doi.org/10.1158/1538-7445.SABCS22-PD13-05

68. Pistilli B, Wildiers H, Hamilton EP, Ferreira AA, Dalenc F, Vidal M, et al. Clinical activity of MCLA-128 (zenocutuzumab) in combination with endocrine therapy (ET) in ER+/HER2-low, non-amplified metastatic breast cancer (MBC) patients (pts) with ET-resistant disease who had progressed on a CDK4/6 inhibitor (CDK4/6i). JCO [Internet]. 2020 May 20 [cited 2022 Nov 21];38(15_suppl):1037–1037. Available from: https://ascopubs.org/doi/abs/10.1200/JCO.2020.38.15_suppl.1037

69. Bardia A, Modi S, Oliveira M, Cortes J, Campone M, Ma B, et al. Phase Ib Dose-escalation/Expansion Trial of Ribociclib in Combination With Everolimus and Exemestane in Postmenopausal Women with HR+, HER2− Advanced Breast Cancer. Clin Cancer Res [Internet]. 2020 Dec 15 [cited 2023 Mar 24];26(24):6417–28. Available from: https://www.ncbi.nlm.nih.gov/pmc/articles/PMC8489181/

70. Rugo HS, Lerebours F, Ciruelos E, Drullinsky P, Ruiz-Borrego M, Neven P, et al. Alpelisib plus fulvestrant in PIK3CA-mutated, hormone receptor-positive advanced breast cancer after a CDK4/6 inhibitor (BYLieve): one cohort of a phase 2, multicentre, open-label, non-comparative study. The Lancet Oncology [Internet]. 2021 Apr 1 [cited 2022 Jul 16];22(4):489–98. Available from: https://www.thelancet.com/journals/lanonc/article/PIIS1470-2045(21)00034-6/fulltext

71. Rugo HS, Lerebours F, Juric D, Turner N, Chia S, Drullinsky P, et al. Abstract PD2-07: Alpelisib + letrozole in patients with PIK3CA-mutated, hormone-receptor positive (HR+), human epidermal growth factor receptor-2-negative (HER2-) advanced breast cancer (ABC) previously treated with a cyclin-dependent kinase 4/6 inhibitor (CDK4/6i) + fulvestrant: BYLieve study results. Cancer Research [Internet]. 2021 Feb 15 [cited 2022 Nov 13];81(4_Supplement):PD2-07. Available from: https://doi.org/10.1158/1538-7445.SABCS20-PD2-07

72. Albanell J, Pérez-García JM, Gil-Gil M, Curigliano G, Ruíz-Borrego M, Comerma L, et al. Palbociclib Rechallenge for Hormone Receptor–Positive/HER-Negative Advanced Breast Cancer: Findings from the Phase II BioPER Trial. Clinical Cancer Research [Internet]. 2023 Jan 1 [cited 2023 Mar 25];29(1):67. Available from: https://www.ncbi.nlm.nih.gov/pmc/articles/PMC9811162/

73. Jhaveri K, Juric D, Yap YS, Cresta S, Layman RM, Duhoux FP, et al. A Phase I Study of LSZ102, an Oral Selective Estrogen Receptor Degrader, with or without Ribociclib or Alpelisib, in Patients with Estrogen Receptor–Positive Breast Cancer. Clinical Cancer Research [Internet]. 2021 Nov 1 [cited 2023 Mar 25];27(21):5760–70. Available from: https://aacrjournals.org/clincancerres/article/27/21/5760/671776/A-Phase-I-Study-of-LSZ102-an-Oral-Selective

74. Lim JSJ, Wong ALA, Ow SGW, Ngoi NYL, Chan GHJ, Ang YLE, et al. Phase Ib/II Dose Expansion Study of Lenvatinib Combined with Letrozole in Postmenopausal Women with Hormone Receptor–Positive Breast Cancer. Clinical Cancer Research [Internet]. 2022 Jun 1 [cited 2023 Mar 26];28(11):2248–56. Available from: https://aacrjournals.org/clincancerres/article/28/11/2248/698933/Phase-Ib-II-Dose-Expansion-Study-of-Lenvatinib

75. Mayer IA, Haley BB, Abramson VG, Brufsky A, Rexer B, Stringer-Reasor E, et al. Abstract PD1-03: A phase Ib trial of fulvestrant + CDK4/6 inhibitor (CDK4/6i) palbociclib + pan-FGFR tyrosine kinase inhibitor (TKI) erdafitinib in FGFR-amplified/ ER+/ HER2-negative metastatic breast cancer (MBC). Cancer Research [Internet]. 2021 Feb 15 [cited 2022 Nov 13];81(4_Supplement):PD1-03. Available from: https://doi.org/10.1158/1538-7445.SABCS20-PD1-03

76. Pimentel I, Roso LL, Vazquez MR, Saenz JÁG, Palacios-Ozores P, Rodriguez JD la H, et al. 262P Niraparib plus aromatase inhibitors (AI) for germinal mutated BRCA1/2 (gBRCAm) or homologous recombination-deficient (HRd), hormone receptor-positive/human epidermal growth factor receptor 2-negative (HR+/HER2-) advanced breast cancer (ABC): LUZERN interim analysis. Annals of Oncology [Internet]. 2022 Sep 1 [cited 2022 Oct 27];33:S657. Available from: https://www.annalsofoncology.org/article/S0923-7534(22)02152-4/fulltext

77. Lim E, Jhaveri KL, Perez-Fidalgo JA, Bellet M, Boni V, Perez Garcia JM, et al. A phase Ib study to evaluate the oral selective estrogen receptor degrader GDC-9545 alone or combined with palbociclib in metastatic ER-positive HER2-negative breast cancer. JCO [Internet]. 2020 May 20 [cited 2022 Nov 21];38(15_suppl):1023–1023. Available from: https://ascopubs.org/doi/abs/10.1200/JCO.2020.38.15_suppl.1023

78. Schwartzberg LS, Bondarenko I, Tolaney SM, Seidman AD, O'Shaughnessy J, Shparyk YV, et al. Results from CONTESSA 2: A multinational, multicenter, phase 2 study of tesetaxel (T) plus a reduced dose of capecitabine (C) in patients (pts) with hormone receptor + (HR+), HER2-metastatic breast cancer (MBC) not previously treated with a taxane. JCO [Internet]. 2021 May 20 [cited 2022 Sep 27];39(15_suppl):1061–1061. Available from: https://ascopubs.org/doi/abs/10.1200/JCO.2021.39.15_suppl.1061

79. O'Shaughnessy J, Schwartzberg L, Piccart M, Rugo HS, Yardley DA, Cortes J, et al. Abstract GS4-01: Results from CONTESSA: A phase 3 study of tesetaxel plus a reduced dose of capecitabine versus capecitabine alone in patients with HER2-, hormone receptor + (HR+) metastatic breast cancer (MBC) who have previously received a taxane. Cancer Research 2021;81:GS4-01. https://doi.org/10.1158/1538-7445.SABCS20-GS4-01.

80. Prat A, Pascual T, Muñoz M, Hernando C, Vazquez S, Blanch S, et al. Abstract P3-06-02: TATEN TRIAL (SOLTI-1716) Targeting non-Luminal disease by PAM50 with pembrolizumab + paclitaxel in Hormone Receptor-positive/HER2-negative (HR+/HER2-) metastatic breast cancer (MBC): interim analysis. Cancer Research [Internet]. 2023 Mar 1 [cited 2023 Mar 26];83(5_Supplement):P3-06-02-P3-06–02. Available from: https://aacrjournals.org/cancerres/article/83/5_Supplement/P3-06-02/717776/Abstract-P3-06-02-TATEN-TRIAL-SOLTI-1716-Targeting

81. Jhaveri KL, Goldman JW, Hurvitz SA, Guerrero-Zotano A, Unni N, Brufsky A, et al. Neratinib plus fulvestrant plus trastzuzumab (N+F+T) for hormone receptor-positive (HR+), HER2-negative, HER2-mutant metastatic breast cancer (MBC): Outcomes and biomarker analysis from the SUMMIT trial. JCO 2022;40:1028–1028. https://doi.org/10.1200/JCO.2022.40.16_suppl.1028.

82. Damodaran S, Plourde PV, Moore HCF, Anderson IC, Portman DJ. Open-label, phase 2, multicenter study of lasofoxifene (LAS) combined with abemaciclib (Abema) for treating pre- and postmenopausal women with locally advanced or metastatic ER+/HER2− breast cancer and an ESR1 mutation after progression on prior therapies. JCO [Internet]. 2022 Jun [cited 2022 Oct 26];40(16_suppl):1022–1022. Available from: https://ascopubs.org/doi/abs/10.1200/JCO.2022.40.16_suppl.1022

83. Tao JJ, Blackford AL, Nunes R, Truica CI, Mahosky J, Jones MK, et al. Abstract P3-01-07: Phase II trial of palbociclib with fulvestrant in individuals with hormone receptor-positive, HER2-negative metastatic breast cancer with disease progression after palbociclib with an aromatase inhibitor. Cancer Research [Internet]. 2023 Mar 1 [cited 2023 Mar 28];83(5_Supplement):P3-01–7. Available from: https://doi.org/10.1158/1538-7445.SABCS22-P3-01-07

84. Bedard P, Mates M, Hilton J, Levasseur N, Awan A, Srikanthan A, et al. Abstract P3-07-10: CCTG IND.236: A Phase 1b trial of combined CFI-402257 and weekly paclitaxel in patients with HER2-negative (HER2-) advanced breast cancer (aBC). Cancer Research [Internet]. 2023 Mar 1 [cited 2023 Mar 28];83(5_Supplement):P3-07–10. Available from: https://doi.org/10.1158/1538-7445.SABCS22-P3-07-10

85. Miller K, Tolaney S, Emens LA, Kim SB, Hamilton E, Saura C, et al. Abstract P4-01-15: Preliminary results from a phase 2 study of praluzatamab ravtansine (CX-2009) in patients with advanced breast cancer (ABC). Cancer Research [Internet]. 2023 Mar 1 [cited 2023 Mar 29];83(5_Supplement):P4-01–15. Available from: https://doi.org/10.1158/1538-7445.SABCS22-P4-01-15

86. Haddad T, D'Assoro A, Suman V, Carter J, McMenomy B, Mayer E, et al. Abstract PD2-05: Randomized phaseII trial to evaluate alisertib alone or combined with fulvestrant for advanced, endocrine-resistant breast cancer (TBCRC 041). Cancer Research 2021;81:PD2-05. https://doi.org/10.1158/1538-7445.SABCS20-PD2-05.

87. Sonnenblick A, Im SA, Lee KS, Tan A, Telli M, Shachar SS, et al. 267P Phase Ib/II open-label, randomized evaluation of second- or third-line (2L/3L) atezolizumab (atezo) + entinostat (entino) in MORPHEUS-HR+ breast cancer (M-HR+BC). Annals of Oncology [Internet]. 2021 Sep 1 [cited 2022 Nov 2];32:S479. Available from: https://www.annalsofoncology.org/article/S0923-7534(21)02779-4/fulltext

88. Goetz MP, Plourde P, Stover DG, Bagegni N, Vidal GA, Brufsky A, et al. LBA20 Open-label, randomized study of lasofoxifene (LAS) vs fulvestrant (Fulv) for women with locally advanced/metastatic ER+/HER2- breast cancer (mBC), an estrogen receptor 1 (ESR1) mutation, and disease progression on aromatase (AI) and cyclin-dependent kinase 4/6 (CDK4/6i) inhibitors. Annals of Oncology [Internet]. 2022 Sep 1 [cited 2022 Oct 27];33:S1387–8. Available from: https://www.annalsofoncology.org/article/S0923-7534(22)03893-5/fulltext

89. Goetz MP, Suman VJ, Reid JM, Kuffel M, Buhrow SA, McGovern RM, et al. Abstract PD7-06: A randomized phase II trial of tamoxifen versus Z-endoxifen HCL in postmenopausal women with metastatic estrogen receptor positive, HER2 negative breast cancer. Cancer Research [Internet]. 2020 Feb 15 [cited 2022 Nov 9];80(4_Supplement):PD7-06. Available from: https://doi.org/10.1158/1538-7445.SABCS19-PD7-06

90. Jimenez MM, Lim E, Gregor MCM, Bardia A, Wu J, Zhang Q, et al. 211MO Giredestrant (GDC-9545) vs physician choice of endocrine monotherapy (PCET) in patients (pts) with ER+, HER2– locally advanced/metastatic breast cancer (LA/mBC): Primary analysis of the phase II, randomised, open-label acelERA BC study. Annals of Oncology [Internet]. 2022 Sep 1 [cited 2022 Sep 25];33:S633–4. Available from: https://www.annalsofoncology.org/article/S0923-7534(22)02101-9/fulltext

91. Mayer EL, Ren Y, Wagle N, Mahtani R, Ma C, DeMichele A, et al. Abstract GS3-06: GS3-06 Palbociclib After CDK4/6i and Endocrine Therapy (PACE): A Randomized Phase II Study of Fulvestrant, Palbociclib, and Avelumab for Endocrine Pre-treated ER+/HER2- Metastatic Breast Cancer. Cancer Research [Internet]. 2023 Mar 1 [cited 2023 Mar 28];83(5_Supplement):GS3-06-GS3-06. Available from: https://aacrjournals.org/cancerres/article/83/5_Supplement/GS3-06/717530/Abstract-GS3-06-GS3-06-Palbociclib-After-CDK4-6i

92. Oliveira M, Pominchuck D, Nowecki Z, Hamilton E, Kulyaba Y, Andabekov T, et al. Abstract GS3-02: GS3-02 Camizestrant, a next generation oral SERD vs fulvestrant in post-menopausal women with advanced ER-positive HER2-negative breast cancer: Results of the randomized, multi-dose Phase 2 SERENA-2 trial. Cancer Research [Internet]. 2023 Mar 1 [cited 2023 Dec 27];83(5_Supplement):GS3-02. Available from: https://doi.org/10.1158/1538-7445.SABCS22-GS3-02

93. Llombart-Cussac A, Harper-Wynne C, Perello A, Hennequin A, Fernandez A, Colleoni M, et al. Second-line endocrine therapy (ET) with or without palbociclib (P) maintenance in patients (pts) with hormone receptor-positive (HR[+])/human epidermal growth factor receptor 2-negative (HER2[-]) advanced breast cancer (ABC): PALMIRA trial. JCO [Internet]. 2023 Jun 1 [cited 2024 Feb 5];41(16_suppl):1001–1001. Available from: https://ascopubs.org/doi/10.1200/JCO.2023.41.16_suppl.1001
