# Supplementary figures and images for: Systematic Review and Network Meta-Analysis on Treating Hormone Receptor-Positive Metastatic Breast Cancer After CDK4/6 Inhibitors
Source: Curr Oncol. 2025 Jan 20;32(1):53. doi: 10.3390/curroncol32010053 (PMC11763720; doi:10.3390/curroncol32010053)

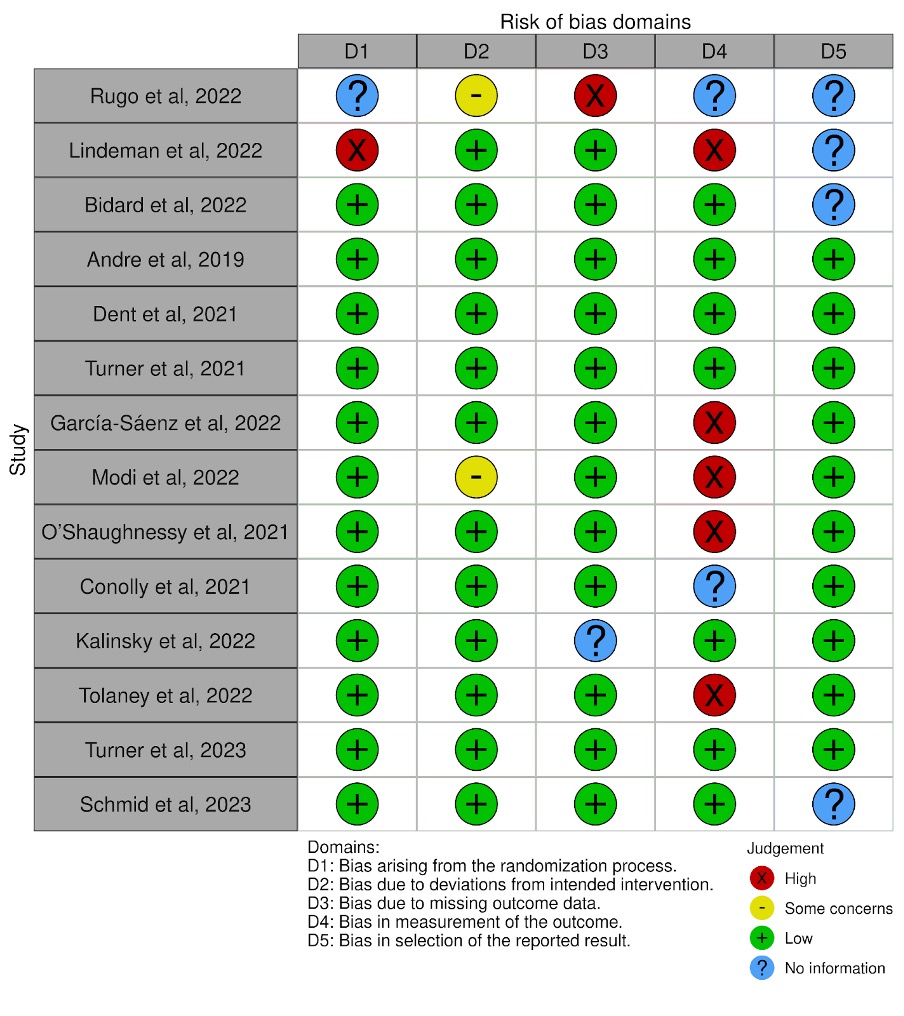

Supplement: Supplementary file 1 [file curroncol-32-00053-s001.zip › Figure S1a.Traffic plot for risk of bias assessment.jpg]

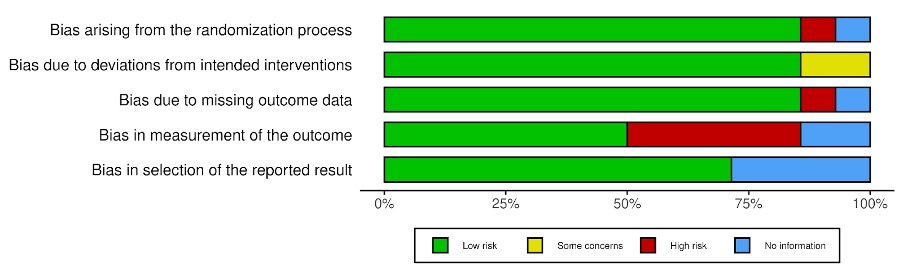

Supplement: Supplementary file 1 [file curroncol-32-00053-s001.zip › Figure S1b.Summary plot for risk of bias assessment.jpg]

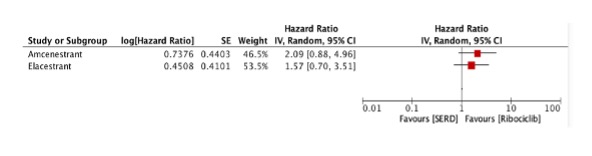

Supplement: Supplementary file 1 [file curroncol-32-00053-s001.zip › Figure S2.Forest plot showing the network meta analysis of progression free survival through network meta-analysis of oral Selective Estrogen Receptor Degraders to continuing ribociclib in mutated ESR1 patients.jpg]

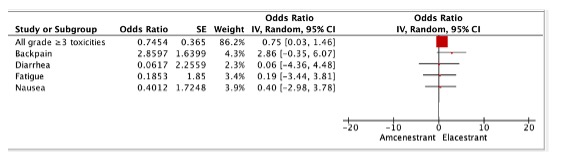

Supplement: Supplementary file 1 [file curroncol-32-00053-s001.zip › Figure S3.Forest Plot showing network meta-analysis of grade 3 and above adverse events for Selective Estrogen Receptor Degraders.jpg]

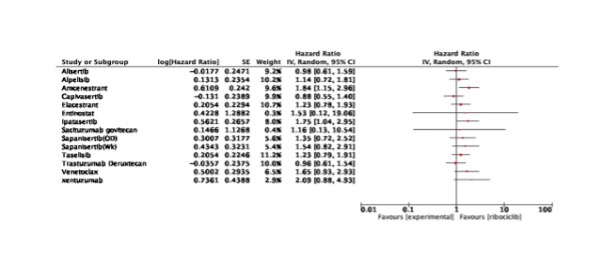

Supplement: Supplementary file 1 [file curroncol-32-00053-s001.zip › Figure S4.Forest Plot showing network meta-analysis of progression free survival of all interventions compared to that of continuing ribociclib.jpg]

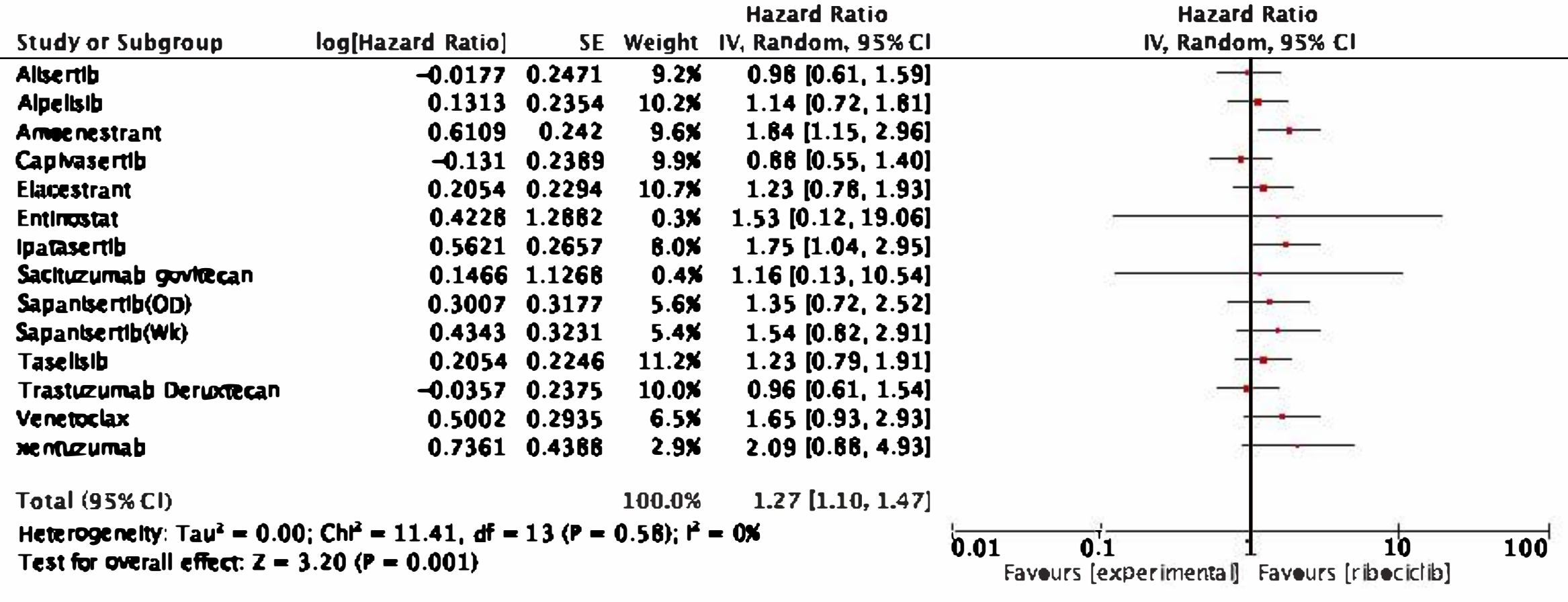

Supplement: Supplementary file 1 [file curroncol-32-00053-s001.zip › Figure S4.Forest Plot showing network meta-analysis of progression free survival of all interventions compared to that of continuing ribociclib.jpg .pdf]

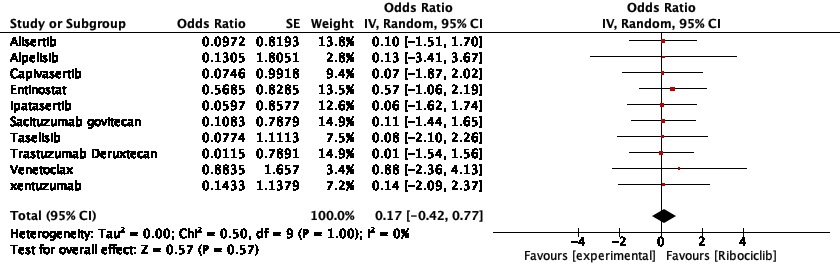

Supplement: Supplementary file 1 [file curroncol-32-00053-s001.zip › Figure S5a.Forest plot of network meta-analysis comparison of Grade≥3 hematological toxicities vs ribociclib.jpg]

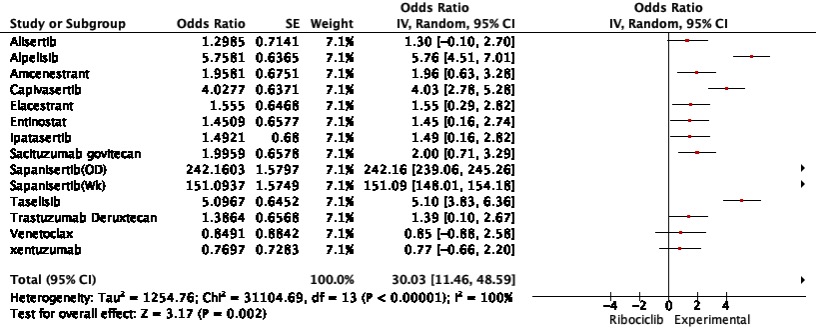

Supplement: Supplementary file 1 [file curroncol-32-00053-s001.zip › Figure S5b.Forest plot of comparison network meta-analysis of Non-hematological grade≥3 adverse effects of all agents vs ribociclib.jpg]
